# Effects of Photon Radiation on DNA Damage, Cell Proliferation, Cell Survival, and Apoptosis of Murine and Human Mesothelioma Cell Lines

**DOI:** 10.1016/j.adro.2022.101013

**Published:** 2022-07-21

**Authors:** Synat Keam, Kelly M. MacKinnon, Rebecca A. D'Alonzo, Suki Gill, Martin A. Ebert, Anna K. Nowak, Alistair M. Cook

**Affiliations:** a National Centre for Asbestos Related Diseases, Institute for Respiratory Health, Perth, Australia; b Medical School, Mathematics and Computing, University of Western Australia, Perth, Australia; c School of Physics, Mathematics and Computing, University of Western Australia, Perth, Australia; d Radiation Oncology, Sir Charles Gairdner Hospital, Perth, Australia; e Radiation Oncology, Sir Charles Gairdner Hospital, 5D Clinics, Claremont, Australia; f Departments of Medical Oncology and Sir Charles Gairdner Hospital, Perth, Australia; g School of Biomedical Sciences, and Mathematics and Computing, University of Western Australia, Perth, Australia

## Abstract

**Purpose:**

To characterize the cellular responses of murine and human mesothelioma cell lines to different doses of photon radiation with a long-term aim of optimizing a clinically relevant in vivo model in which to study the interaction of radiation therapy and immunotherapy combinations.

**Methods and Materials:**

Two murine mesothelioma cell lines (AB1 and AE17) and 3 human cell lines (BYE, MC, and JU) were used in the study. Cells were treated with increasing doses of photon radiation. DNA damage, DNA repair, cell proliferation, and apoptosis at different time points after irradiation were quantified by flow cytometry, and cell survival probability was examined using clonogenic survival assay.

**Results:**

DNA damage increased with escalating dose in all cell lines. Evident G2/M arrest and reduced cell proliferation were observed after irradiation with 8 Gy. DNA repair was uniformly less efficient at higher compared with lower radiation-fraction doses. The apoptosis dose response varied between cell lines, with greater apoptosis observed at 16 Gy with human BYE and murine AB1 cell lines but less for other studied cell lines, regardless of dose and time. The α/β ratio from the cell survival fraction of human mesothelioma cell lines was smaller than from murine ones, suggesting human cell lines in our study were more sensitive to a change of dose per fraction than were murine mesothelioma cell lines. However, in all studied cell lines, colony formation was completely inhibited at 8 Gy.

**Conclusions:**

A threshold dose of 8 Gy appeared to be appropriate for hypofractionated radiation therapy. However, the radiation therapy doses between 4 and 8 Gy remain to be systematically analyzed. These observations provide an accurate picture of the in vitro response of mesothelioma cell lines to photon irradiation and characterize the heterogeneity between human and murine cell lines. This information may guide in vivo experiments and the strengths and limitations of extrapolation from murine experimentation to potential human translation.

## Introduction

Malignant pleural mesothelioma (MPM) is an aggressive cancer of the pleura, predominantly arising from asbestos exposure.^1^ Patients with MPM have an extremely poor prognosis, with a median survival of 9 to 12 months.^2^ From 2003 until very recently, the standard first-line therapy for mesothelioma was cisplatin and pemetrexed chemotherapy; however, this combination only modestly improves patient survival.^3^ Recently, immunooncology and antiangiogenic agents have begun to challenge this standard of care. In 2021, results from the CheckMate 743 trial showed a benefit from treatment with the immune checkpoint inhibitors ipilimumab and nivolumab compared with combination chemotherapy, particularly in patients with nonepithelioid disease.^4^ The vascular endothelial growth factor inhibitor bevacizumab showed increased survival of more than 2 months when added to chemotherapy but has not been adopted into standard-of-care treatment.^5^ Localized radiation therapy (RT) is used for multiple purposes in treating mesothelioma, including treatment of procedure tract metastases, adjuvant hemithoracic radiation as part of aggressive multimodality therapy, and as palliative therapy to individual lesions.^6^ However, high-dose RT for local control can have toxic effects and does not palliate symptoms in a wholly effective manner.^7^, ^8^, ^9^

As demonstrated in preclinical models in other cancer types,^10^, ^11^, ^12^ RT can be combined with immune checkpoint blockade to enhance treatment efficacy owing to the ability of RT to generate immunogenic cell death,^13^ activate the cGAS-Sting pathway (type I interferon),^14^ increase intratumoral cytotoxic T lymphocytes (CD8^+^),^15^^,^^16^ decrease myeloid-derived suppressor cells,^17^ and normalize tumor blood vessels.^18^, ^19^, ^20^, ^21^, ^22^ Synergy, increased survival, and even abscopal effects from combinations of RT and immunotherapy (using agents such as anti-PD1/PD-L1 and anti-CTLA-4) have been reported in several mouse studies including in breast cancer,^11^ intracranial glioma,^12^ colon adenocarcinoma,^17^ *KRAS*-mutant lung cancer,^23^ and lymphoma plus Lewis lung carcinoma.^24^ With these encouraging outcomes from preclinical studies, many clinical trials are currently investigating radioimmunotherapy combinations in different cancer types, with promising preliminary outcomes.

In a recent phase 2 clinical trial in early-stage non-small cell lung cancer, 60 patients were randomly assigned to receive either durvalumab alone or durvalumab plus stereotactic body radiation therapy (8 Gy × 3); pathologic responses were 53.3% (95% CI, 34.3%-71.7%) versus 6.7% (95% CI, 0.8%-22.1%), respectively, and several complete pathologic responses were observed in the combination group.^25^ In a retrospective study examining the efficacy of hypofractionated RT plus ipilimumab (Ipi-RT) compared with ipilimumab alone in melanoma brain metastases, an increased median overall survival of 19 months was observed in the Ipi-RT arm compared with 10 months in the ipilimumab arm (*P* = .001), and Ipi-RT had a significantly higher complete response rate than ipilimumab did alone (25% vs 6.5%). Several other studies have reported an abscopal response in patients with advanced melanoma after RT plus ipilimumab^26^^,^^27^ and increased survival after RT and immunotherapy.^28^, ^29^, ^30^

For mesothelioma, however, the benefits of rRT and immunotherapy are currently understudied in both the clinical and preclinical settings. To date, 1 mouse study using the AB12 mesothelioma model to examine the effects of radioimmunotherapy demonstrated an increased T-cell influx into primary and secondary tumors after hypofractionated RT, with a highly activated CD8^+^ T-cell phenotype observed after the addition of anti-CTLA-4. Delayed tumor growth in the secondary tumor after irradiation to the primary tumor compared with the sham-RT group was also observed. The authors also reported increased expression of genes such as interferon-γ, granzyme B, ICOS, CD80, and CD86 after RT plus anti-CTLA-4.^31^

Owing to the paucity of data from the clinic in combining RT and immunotherapy in mesothelioma, it is important to study this approach in a robust animal model.^32^^,^^33^ However, to understand the utility of the animal model, it is vital to first characterize the radiation response of both murine and human mesothelioma cell lines, because this work may enable assessment of the suitability of mouse cell lines and identification of the approximate radiation dose to combine with immunotherapy for mesothelioma. As of 2020, according to the National Cancer Institute's clinical trial and evaluation program, a dose-fractionation schedule (8 Gy × 3) is considered a standard fractionation for combination with immunotherapy.^34^ However, each cancer type is different in its biology and microenvironment. Therefore, characterizing the in vitro radiation response may help guide optimal selection of doses to combine with immunotherapy in animal models.

The effects of RT are exerted primarily through damage to cellular DNA.^35^ Ordinarily, DNA damage will activate DNA repair machinery and arrest the cell cycle to allow repairs to be carried out, therefore restoring genome integrity.^36^ If cells are capable of repairing the DNA damage, they will resume normal cycling. However, if the damage is too extensive, cells may progress to cell death, or senescence.^37^ Clinical reports suggest that mesothelioma is relatively resistant to RT.^38^ However, in vitro studies revealed human mesothelioma cell lines were sensitive to conventional doses of radiation.^39^^,^^31^ Given the complex biological effects of RT delivered with variable dose and fractionation schedules, our present study investigated cellular damage in multiple human and murine mesothelioma cell lines after radiation by incorporating several outcome measures (including DNA damage, cell cycle, cell proliferation, survival, and apoptosis) at varying doses of photon radiation. In this article, we characterize the in vitro biological responses to different doses of radiation and identify the optimal radiation dose capable of inducing antitumor immune responses (particularly via DNA damage and clonogenic survival assay with characterization of the survival curve via the α/β ratio) for in vivo radioimmunotherapy studies. In addition, we compare the biological responses of murine and human mesothelioma cell lines to radiation, thereby informing the suitability of the murine cell lines as preclinical models and selecting the optimal dose for future studies.

## Materials and Methods

### Cell lines

Murine mesothelioma cell lines (AB1 and AE17) were generated as previously described.^40^^,^^41^ They were grown and maintained in complete RPMI medium containing 2 mM glutamine, 20 mM N-2-hydroxyethylpiperazine-N′-2-ethanesulfonic acid (Sigma Aldrich, Australia), 100 U/mL benzylpenicillin, 500 mg/mL gentamicin, 0.05 mM 2-mercapteothanol, and 10% neonatal calf serum (NCS). Human mesothelioma cell lines JU77 (JU), MC_P5001 (MC), and BYE10412 (BYE) were generated from patient pleural effusions and sourced from the National Centre for Asbestos Related Diseases Biobank (Perth, Australia). The cell lines were maintained in RPMI medium containing 2 mM glutamine, 20 mM N-2-hydroxyethylpiperazine-N′-2-ethanesulfonic, 0.05 mM 2-mercaptoethanol, and 5% fetal calf serum. The characteristics of the cell lines are summarized in supplementary Table E1.

### Irradiation

Exponentially growing mesothelioma cell lines were irradiated in 50-mL tubes (0.5 × 10^6^ cells/mL) at room temperature, using a Gammacell irradiator 3000 SN 211 (Nordion, Ottawa, Canada) at various doses as specified. The dose rate from the cesium-137 source was 3.87 Gy/min. The unit was calibrated according to National Institute of Standards and Technology standards.

### Cell proliferation

Assessment of cell proliferation after photon radiation exposure was done by pulse labeling the irradiated cells with 5-bromo-2′-deoxyuridine (BrdU), followed by quantification of the proportion of BrdU^+^ cells by flow cytometry. In brief, cells were centrifuged (300 relative centrifugal force (RCF), 3 minutes) to remove the irradiated media immediately after radiation. Supernatants were discarded, and cell pellets were resuspended in fresh RPMI medium; cells (0.5 × 10^6^) were transferred to a T175 flask and incubated at 37°C in a humidified 5% CO_2_ atmosphere. BrdU (20 µg) was added to the appropriate flask for the last hour of incubation, and cells were harvested at either 1, 6, 24, 48, or 72 hours after radiation exposure.

### Cell fixation and storage

One hour after exposure, BrdU pulse labeling was completed at each time point. Cell culture medium was transferred from flasks to 50-mL tubes. The flasks were washed with 10 mL 1X phosphate-buffered saline (PBS), and 2 mL warm trypsin was added to dislodge adherent cells, which were incubated for 2 minutes at 37°C in a humidified 5% CO_2_ atmosphere. The cells were washed by resuspension in 10 mL culture media to deactivate trypsin and centrifuged (300 RCF, 3 minutes). Supernatants were discarded, and cell pellets were resuspended in 1 mL culture media. Cell counts were performed using trypan blue exclusion; 0.5 × 10^6^ cells were collected and washed twice with 2 mL 1X PBS and centrifuged (300 RCF, 3 minutes). Supernatants were discarded, and cell pellets were fixed with 100 µl Cytofix/Cytoperm Fixation and Permeabilization solution (BD Biosciences, Franklin Lakes, New Jersey) for 20 minutes on ice. After cell fixation, cells were washed with staining buffer (1X PBS, 2% NCS), transferred into 1 mL freezing medium (10% dimethyl sulfoxide, 90% NCS), and stored at –80°C until staining.

### Flow cytometry

Flow cytometry staining was performed using “apoptosis, DNA damage, and cell proliferation” kits (BD Biosciences, Franklin Lakes, New Jersey) according to the manufacturer's instructions. In brief, cells frozen at –80°C were thawed at room temperature and transferred to corresponding labeled staining tubes. Cells were washed twice with 2 mL staining buffer and centrifuged (300 RCF, 3 minutes). Supernatants were discarded, and cell pellets were fixed with 100 µL BD Cytofix/Cytoperm Fixation and Permeabilization solution for 20 minutes on ice. After fixation, cells were washed with 500 µL 1X PermWash from the BD Cytofix/Cytoperm kit by centrifugation (300 RCF, 3 minutes). Supernatants were discarded, and cell pellets were treated with 100 µL deoxyribonuclease (300 µg/mL) in sterile 1X PBS to expose BrdU epitopes, then incubated for 1 hour at 37°C in a humidified 5% CO_2_ atmosphere. After deoxyribonuclease treatment, the cells were washed with 500 µL 1X PermWash. Supernatants were discarded, and cell pellets were stained using 20 µL antibody cocktail—mouse anti-BrdU-PerCP-C5.5 (Cat # 51-9007682, Clone: 34D, dilution: 1/40), mouse anti-γ-H2AX-AF467 (Cat # 560447, Clone: N1-431, dilution: 1/10), and mouse anticleaved PARP-PE (Cat # 51-9007684, Clone: F21-852, dilution: 1/40)—diluted in staining buffer, then incubated in the dark for 20 minutes at room temperature. After staining, cells were washed with 500 µL 1X PermWash by centrifugation (300 RCF, 3 minutes). Cell pellets were then stained with DAPI (Cat # 564907, dilution: 1/1000) for 15 minutes. After staining, cells were washed with 1 mL staining buffer and resuspended in 200 µL staining buffer. All staining tubes were acquired on BD LSRFortessa (BD Biosciences), with 100,000 events recorded per sample. Data were analyzed using Flowjo_V10 (Treestar, Oregon).

### Clonogenic survival assay

Exponentially growing cells were irradiated (1-8 Gy) in 50-mL tubes, then seeded into T25 flasks in triplicate. Flasks were incubated at 37°C in a humidified 5% CO_2_ atmosphere, and the media were changed every 3 days. Fifteen days after irradiation, cells were fixed with 100% methanol for 5 minutes and stained with 0.01% crystal violet. Colonies comprising 50 cells or more were counted using ColCount (Oxford Optronix, Abingdon, United Kingdom). According to the linear quadratic model, the survival fraction (*SF*) is mathematically related to dose *D*, using the relationship

$$SF=e^{-\alpha D-\beta D^{2}}$$

where α and β are constants for a particular tumor or normal tissue that represent the linear and quadratic components of cell kill, respectively. The α/β ratio is indicative of the sensitivity of that cell line to changes in the dose per fraction.^42^ The conventional dose per fraction in cancer treatment is 2 Gy per day. Hypofractionation describes treatment delivered at a schedule larger than 2 Gy per day. Tumors with a small α/β ratio (for example, an α/β ratio of 2) are more sensitive to larger doses per fraction (hypofractionation) and are killed more effectively; thus, the total dose maybe lowered if hypofractionation is used. Tumors with a high α/β ratio (for example, an α/β ratio of 10) indicate cells are relatively less sensitive to dose per fraction; thus, they need a higher total dose instead to achieve effective tumor kill.^43^ Therefore, determining the α/β ratio of a particular mesothelioma cell line is vitally important for future studies in which RT is used to be able to compare results.

### Statistical analysis

Data were analyzed and visualized using the R environment for statistical computing (version 4.0.5). Multiple group comparisons were performed using 1-way analysis of variance with Tukey adjustment. The analysis of covariance was used to compare group means in a multiple linear regression model for DNA repair with Tukey adjustment. A 4-parameter log-logistic regression model for DNA damage dose response was fitted through the relationship

$$f\left(x,\left(b,c,c,e\right)\right)=c+\frac{d-c}{(1\;+\;\text{exp}\left(b\left(\log\left(x\right)-\log\left(e\right)\right)\right)}$$

where *b* (slope), *c* (lower limit), *d* (upper limit, and *e* (ED50, or 50% DNA damage) are fitting parameters.^44^ Based on the exploration of the model, cell proliferation dose response was fitted using a generalized linear least square because it allowed us to examine the different variance structures for cell line strata. We also considered a log-transformation of the response variable (BrdU), because this improved the model fit. The model formula is explained through the following relationship:

$$BrdU_{di}=\;a+\;\beta 1\times Dose_{d}\;+\beta 2\times Cell_{i}+\;\beta 3\times Cell_{i}\times Dose_{d}+\varepsilon i,$$

where *BrdU_di_* is the outcome at dose *d* (0, 2, 8, or 16) on the *_i_*th cell (AB1, AE17, BYE, JU, and MC) and depends on the β parameters in linear fashion; α, β1, β2, and β3 represent the fixed intercept (fixed effects of dose, cell, and dose–/–cell interaction, respectively); and *εi* is the overall error term in the model. Data are presented as the mean ±1 standard deviation unless otherwise stated, and the experiment was independently repeated at least 3 times.

## Results

### Increased DNA damage responses after photon radiation exposure were proportional to doses

**Figure 1:**
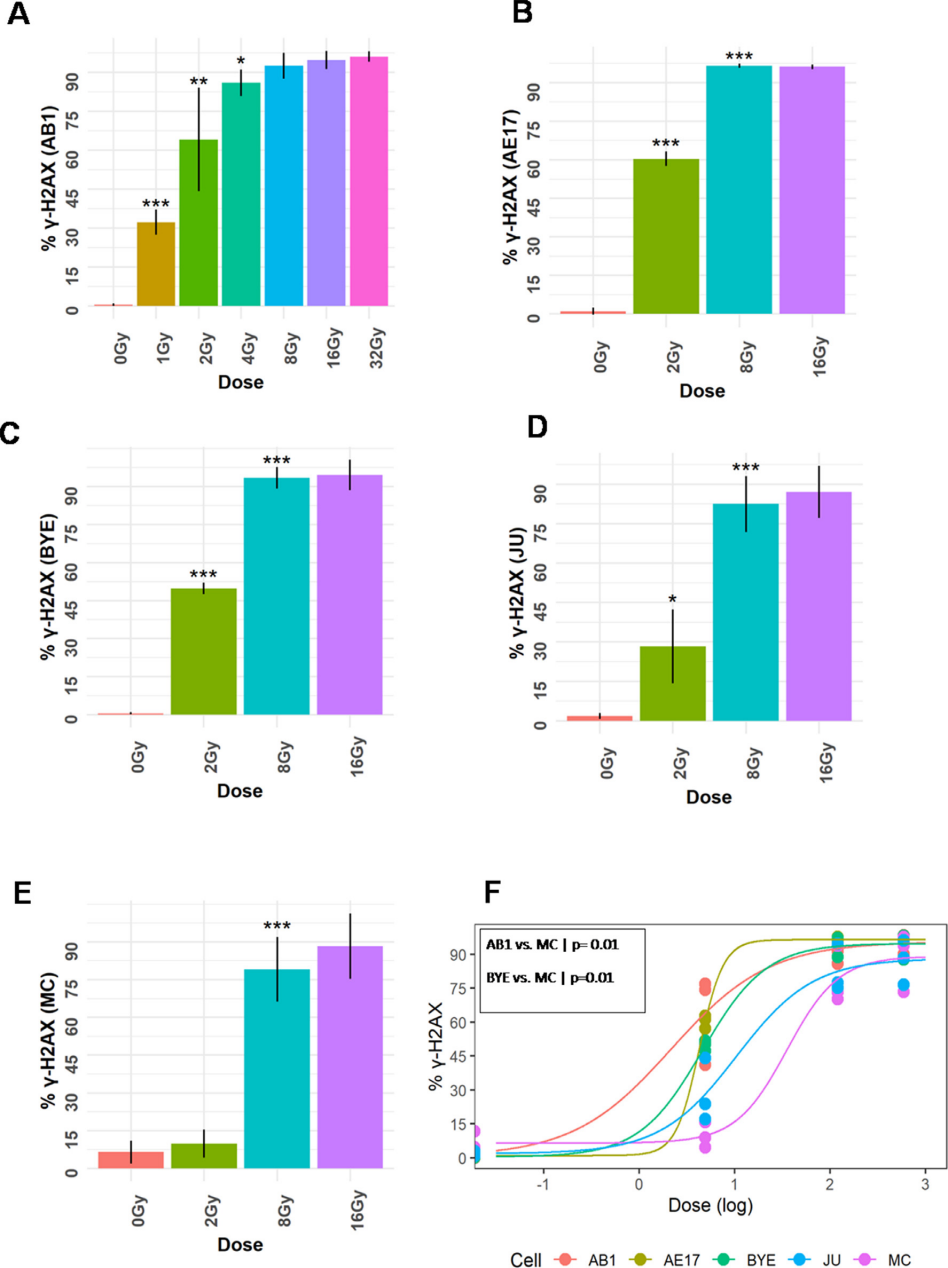
DNA damage increases as a function of dose. Murine and human mesothelioma cell lines were treated with increasing doses of photon radiation, whereupon DNA damage responses (γ-H2AX) were quantified by flow cytometry. A-E, DNA damage responses of AB1, AE17, BYE, JU, and MC cell lines, respectively. F, Differences in DNA damage responses specifically with an ED50 (50% DNA damage) parameter between BYE and MC cell lines and AB1 and MC cell lines. One-way analysis of variance for multiple group comparison with Tukey adjustment for pairwise comparison. A 4-parameter log-logistic multiple regression model for DNA damage dose-response analysis. *P < .05; **P < .01; ***P < .001.

We first examined DNA damage response using the level of γ-H2AX at 1 hour after radiation. The flow cytometry gating strategy for γ-H2AX is demonstrated in supplementary Fig. E1. The level of γ-H2AX increased as the radiation dose increased, reaching a maximum at approximately 8 Gy for the AB1, AE17, BYE, and JU cell lines (Fig. 1A-D). Interestingly, the γ-H2AX level of the MC cell line did not differ between radiation doses of 0 Gy and 2 Gy, indicating that a significant DNA damage response was not induced by 2 Gy. However, the level of γ-H2AX significantly increased at 8 Gy (Fig. 1E). Comparison of the DNA damage responses of the murine and human mesothelioma cell lines demonstrated a difference in ED50 (50% DNA damage) compared with the AB1 and MC cell lines (*P* = .01; Fig. 1F and Table E2) and between the BYE and MC cell lines (*P* = .01; Fig. 1F and Table E2). There was no difference in slope or lower and upper limit parameters among all the studied cell lines.

### High-dose radiation arrested the cell cycle at G2/M phase in the murine but not the human mesothelioma cell line

**Figure 2:**
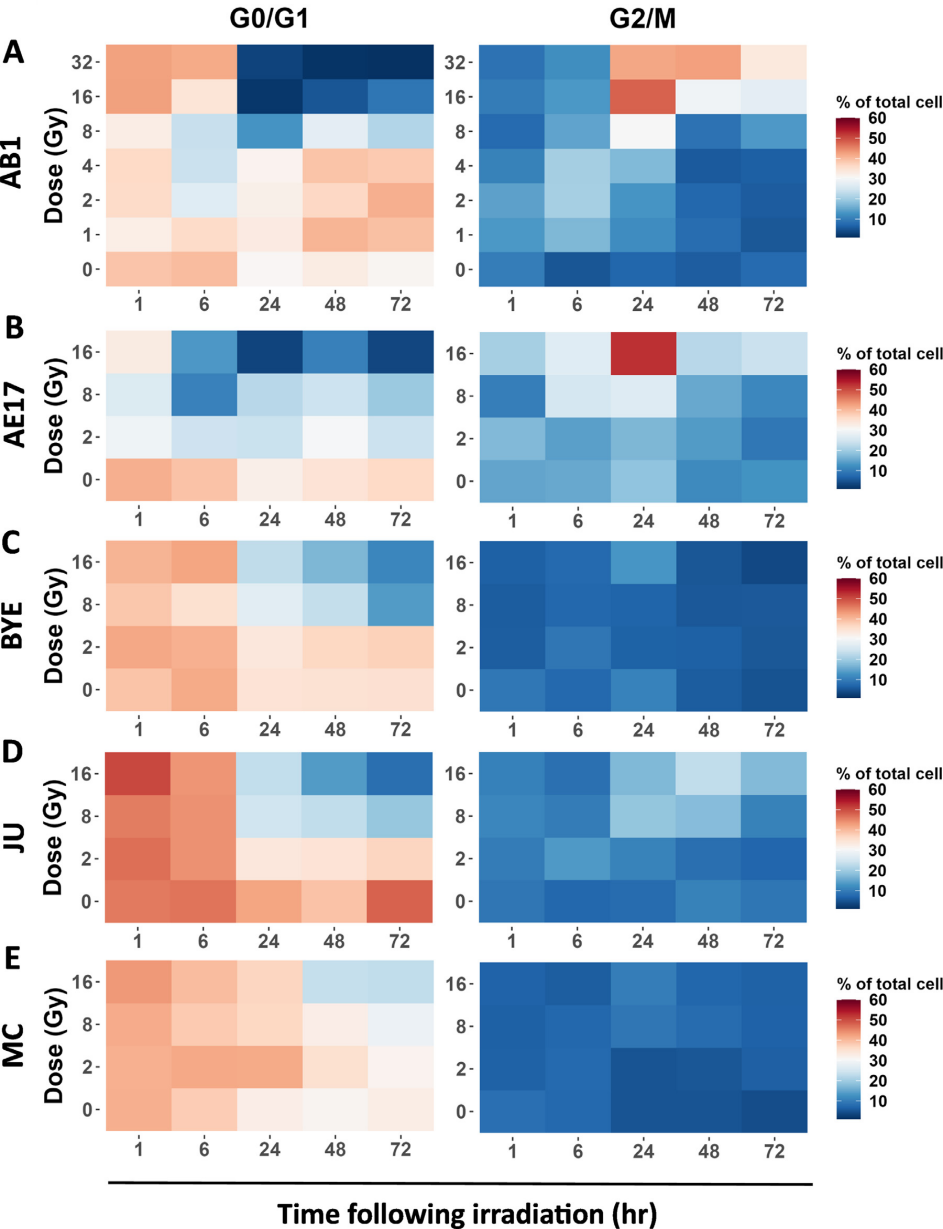
High-dose photon radiation arrests the cell cycle of murine cell lines at the G2/M phase. A, B, G2/M phase arrest of the AB1 and AE17 cell lines after 24 hours at 8, 16, and 32 Gy. C-E, cell-cycle pattern of BYE, JU, and MC cell lines. Data are generated from 3 independent repeats.

DNA damage induces cell-cycle arrest at specific checkpoints for cells to repair DNA.^45^ Therefore, we next assessed the effects of photon radiation on the cell cycle of mesothelioma cell lines through the examination of DNA content (Fig. E2). All pairwise comparisons of the cell cycle for each dose at 24, 48, and 72 hours are summarized in Table E3. Cell cycle arrest at G2/M was not observed at the earliest time points (1 and 6 hours) after irradiation, regardless of dose, in any cell line studied (Fig. 2A-E). However, we observed a reduction in G0/G1 at 1 hour and 6 hours between 0 Gy and 8 Gy (Fig. 2B and Table E3). Doses of 1, 2, and 4 Gy did not significantly change the proportion of cells in G0/G1 and G2/M compared with 0 Gy at any time point after irradiation in any cell line (Fig. 2A-E). However, a decreasing proportion of cells in G0/G1 was observed in AB1 cells, reaching a nadir 24 hours after irradiation with 8 Gy compared with untreated cells (*P* = .008; Table E3A and Fig. 2A). This was paralleled by an increase in G2/M arrest (Fig. 2A). Moreover, the proportion of the G0/G1 and G2/M population of AB1 cells returned to normal levels at 48 and 72 hours, indicating that the cell-cycle arrest at the G2/M phase after 8-Gy irradiation was temporary (Fig. 2A). Conversely, doses of 16 and 32 Gy comprehensively arrested the cell cycle at G2/M, with a significant proportional increase in the G2/M population and decrease in G0/G1 cells at 24, 48, and 72 hours compared with untreated cells (Table E3A and Fig. 2A).

A temporary cell-cycle arrest was also observed in the AE17 cell line after 8-Gy irradiation, because the level of G2/M was significantly higher than in untreated cells at 24 hours (*P* = .01; Table E3B and Fig. 2B). Additionally, a significant reduction in the G0/G1 population compared with the untreated group was also observed at 16 Gy from 24 hours (Table E3B and Fig. 2B). However, G2/M arrest was only observed at 24 and 72 hours with 16 Gy (Table E3B and Fig. 2B); the G2/M population did not differ from the untreated group at 48 hours (Table E3B and Fig. 2B).

We also observed a decreased proportion of the G0/G1 population in the BYE and JU cell lines at 72 hours at 8 and 16 Gy compared with 0 Gy (Table E3C-D and Fig. 2C-D). However, the G2/M arrest was not observed with these 2 cell lines (Fig. 2C-D). For the MC cell line, the proportion of G0/G1 appeared lower at 16 Gy from 48 hours. However, it did not reach significantly different levels compared with the untreated group, and the proportion of G2/M did not change regardless of dose and time (Fig. 2E and Fig. E3E).

### DNA repair was different between low and higher doses but similar between murine and human mesothelioma cell lines

**Figure 3:**
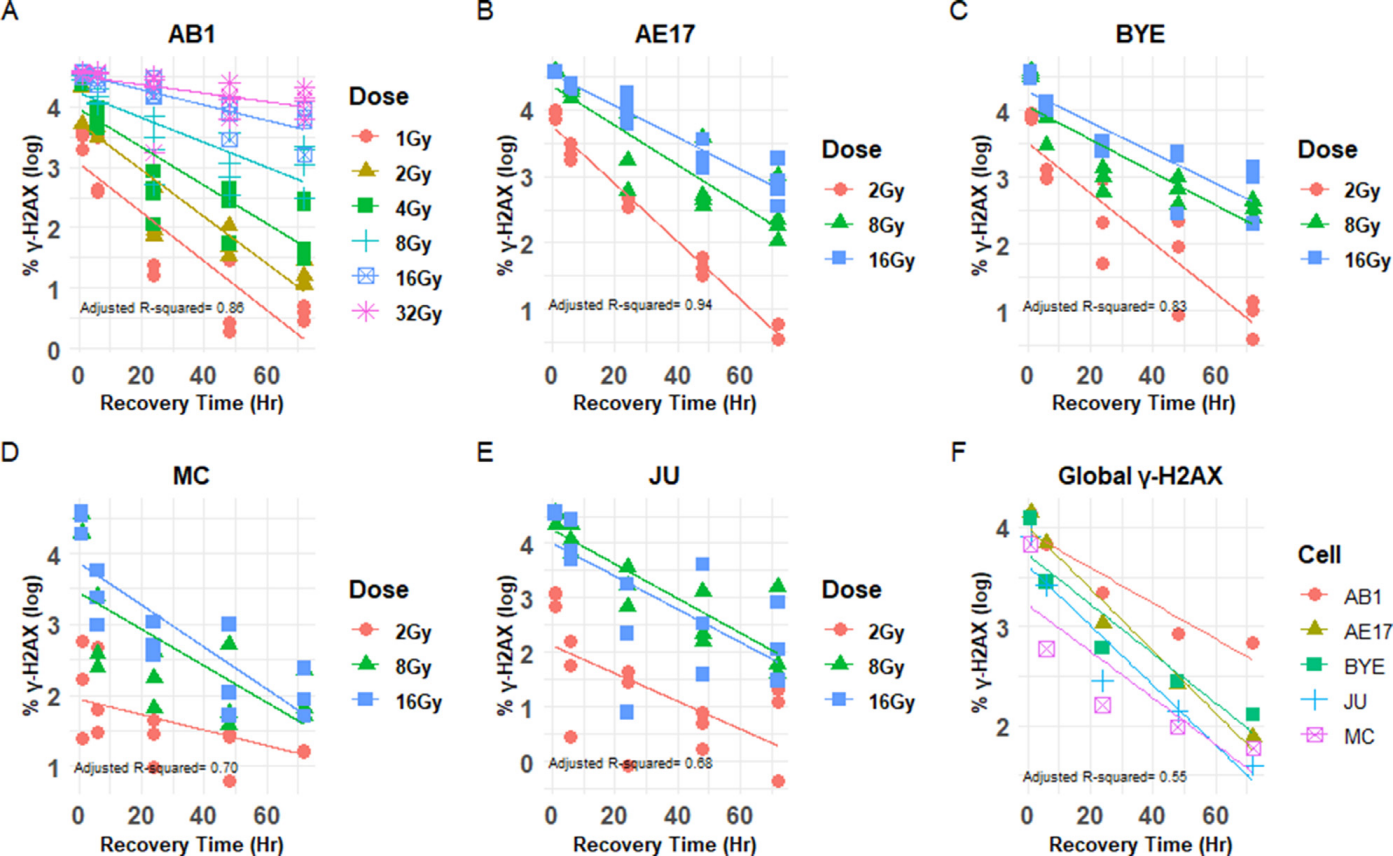
Global DNA repair responses after radiation therapy are similar for both murine and human mesothelioma cell lines. A-E, DNA repair of AB1, AE17, BYE, MC, and JU cell lines, respectively, after receiving different doses of radiation. F, A comparison of global DNA repair of murine and human mesothelioma cell lines. Analysis of covariance for multiple linear regression model with interaction using Tukey adjustment: the linear model for global γ-H2AX with interaction was visualized as a mean. Adjusted *R*^2^ is one of the indications of goodness-of-fit of the final model.

Rapid reduction in γ-H2AX expression over time after irradiation suggests efficient DNA repair.^46^^,^^47^ To examine DNA repair efficiency of murine and human mesothelioma cell lines, the decrease in γ-H2AX over time after irradiation was modeled for each dose and all studied cell lines. Pairwise comparisons of DNA-repair regression slopes between different doses of the AB1 cell line are summarized in Table E4. We observed a rapid drop in the level of γ-H2AX in the AB1 cell line at 1 Gy, and the slope did not differ from those of 2 and 4 Gy (Table E4 and Fig. 3A). However, the level of γ-H2AX remained higher during a 72-hour period at 8 Gy and was different from 1 Gy (*P* = .01; Table E4 and Fig. 3A). This pattern persisted at 16 and 32 Gy, with regression slopes significantly different from 1, 2, and 4 Gy (Table E4 and Fig. 3A), suggesting that the DNA repair machinery of the AB1 cell line was less efficient at doses higher than 8 Gy. Another murine cell line, AE17, had a similar DNA repair pattern to AB1, with a rapid decrease in γ-H2AX expression at 2 Gy, but not for higher doses of 8 and 16 Gy. A significant difference in the γ-H2AX slope was seen between 2 Gy and the higher doses of 8 and 16 Gy (Table E4 and Fig. 3B). This pattern was also observed for the BYE, JU, and MC human cell lines (Table E4 and Fig. 3C-E). Moreover, there was no significant difference in global DNA repair in either the murine or the human mesothelioma cell lines after irradiation with 2, 8, and 16 Gy (Fig. 3F), because the slopes between cell lines were not significantly different.

### Inhibition of cell proliferation peaked at 8 Gy and was similar between murine and human mesothelioma cell lines

**Figure 4:**
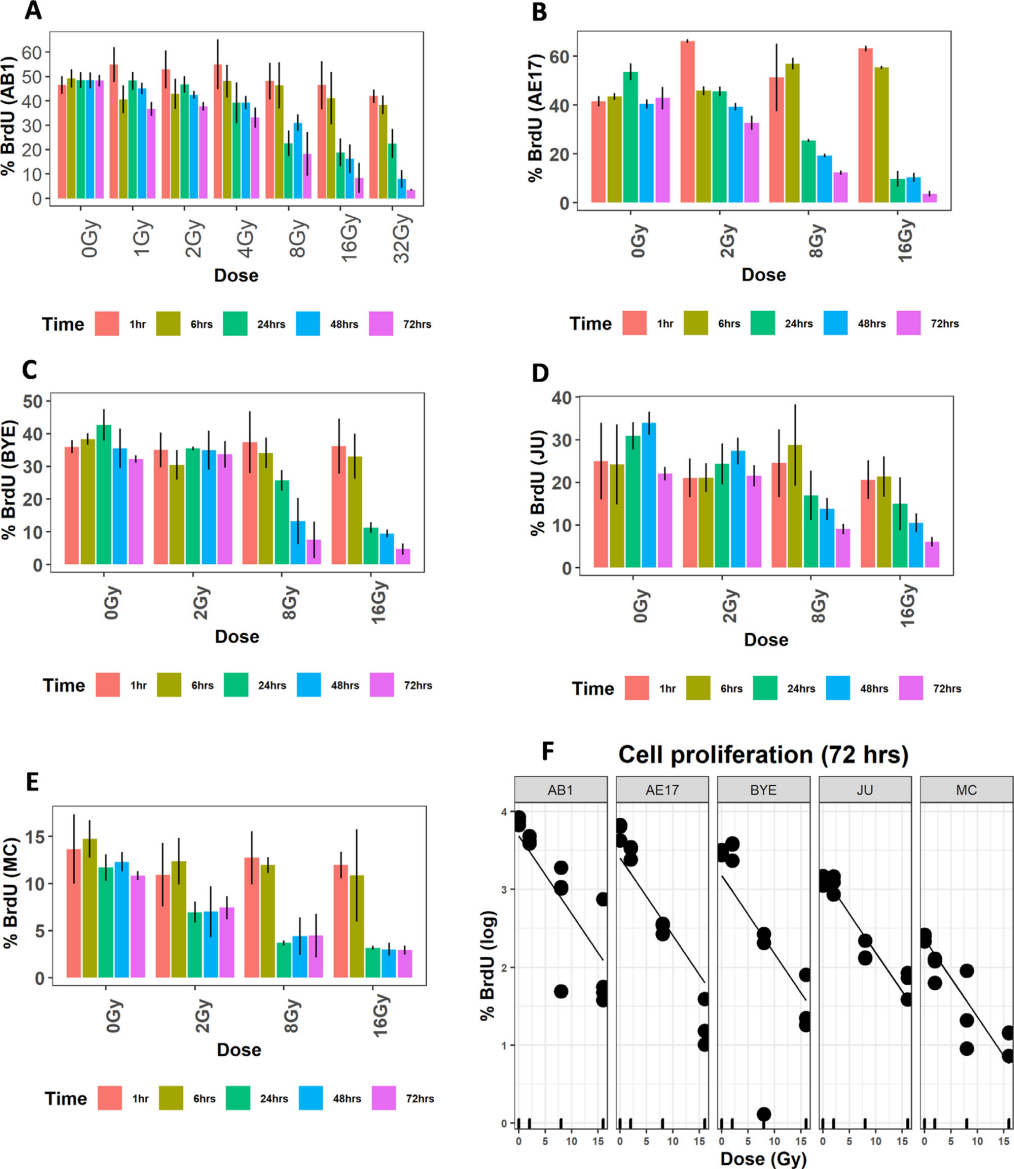
Cell proliferation is greatly inhibited by 8-Gy radiation. A-E, Cell proliferation of AB1, AE17, BYE, JU, and MC cell lines, respectively. F, Cell proliferation of the murine and human cell line after exposing to 0, 2, 8, and 16 Gy at 72 hours, fitted using multiple generalized linear least square regression.

We observed no change in the pattern of cell proliferation, compared with unirradiated cells, in any of our cell lines at any postirradiation time point after treatment with 1, 2, or 4 Gy (Fig. E3 and Fig. 4A-E). Interestingly, a decrease in cell proliferation was not observed at 1 or 6 hours regardless of radiation dose, suggesting a minimum 6-hour delay in the inhibitory effects of photon radiation (Fig. 4A-E). However, a decrease in cell proliferation was observed 24 hours after irradiation with 8 Gy for both murine and human cell lines (Fig. 4A-E). The distinct reduction in cell proliferation was seen at higher doses of 16 and 32 Gy for the AB1 (Fig. 4A) and 16 Gy for the AE17, BYE, JU, and MC cell lines from 24 hours (Fig. 4B-E). Among all postirradiation time points, inhibition of cell proliferation peaked after 72 hours; this time point was therefore used to examine the dose-response relationship and compare cell proliferation patterns between murine and human mesothelioma cell lines. Overall, there was a significant association between radiation dose and reduction in cell proliferation (Fig. 4F). However, cell proliferation did not differ between cell lines at 72 hours, because the slopes were similar (Fig. 4F).

### Human mesothelioma cell lines were more sensitive than mouse cell lines to a change of dose per fraction

**Figure 5:**
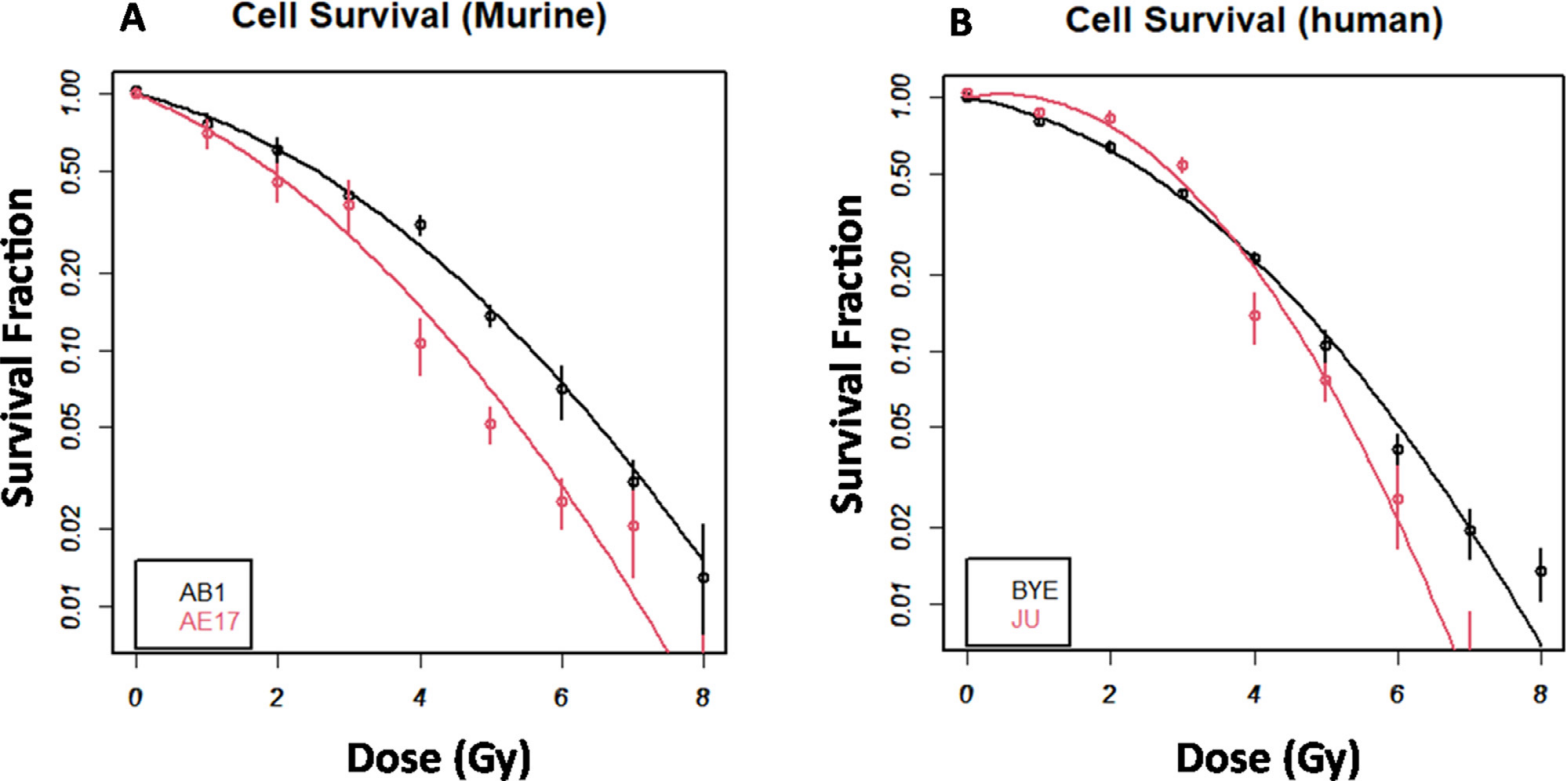
Decreasing cell-survival fraction of murine and human mesothelioma cell lines. A, Survival fraction of murine, AB1, and AE17 and B, survival fraction of human mesothelioma cell lines and BYE and JU cell lines.

To examine the sensitivity of our cell lines to a change of dose per fraction, we performed a colony formation assay to allow accurate calculation of the α/β ratio (Fig. E4 and Table E5). Our data indicated the α/β ratios for AB1 and AE17 were similar: 3.34 and 4.79, respectively (Fig. 5A and Table E5). For human cell lines BYE and JU, the α/β ratios were 1.76 and 0.97, respectively (Table E5 and Fig. 5B). The α/β ratio of murine mesothelioma cell lines was higher than that of human cell lines, indicating that human cell lines exhibit a greater increase in response to increasing the dose per fraction than do murine cell lines. The MC cell line is extremely slow growing and did not form colonies. Therefore, this cell line was not used for the experiment.

### Radiation induced varying levels of apoptosis in a dose- and cell line–dependent manner

**Figure 6:**
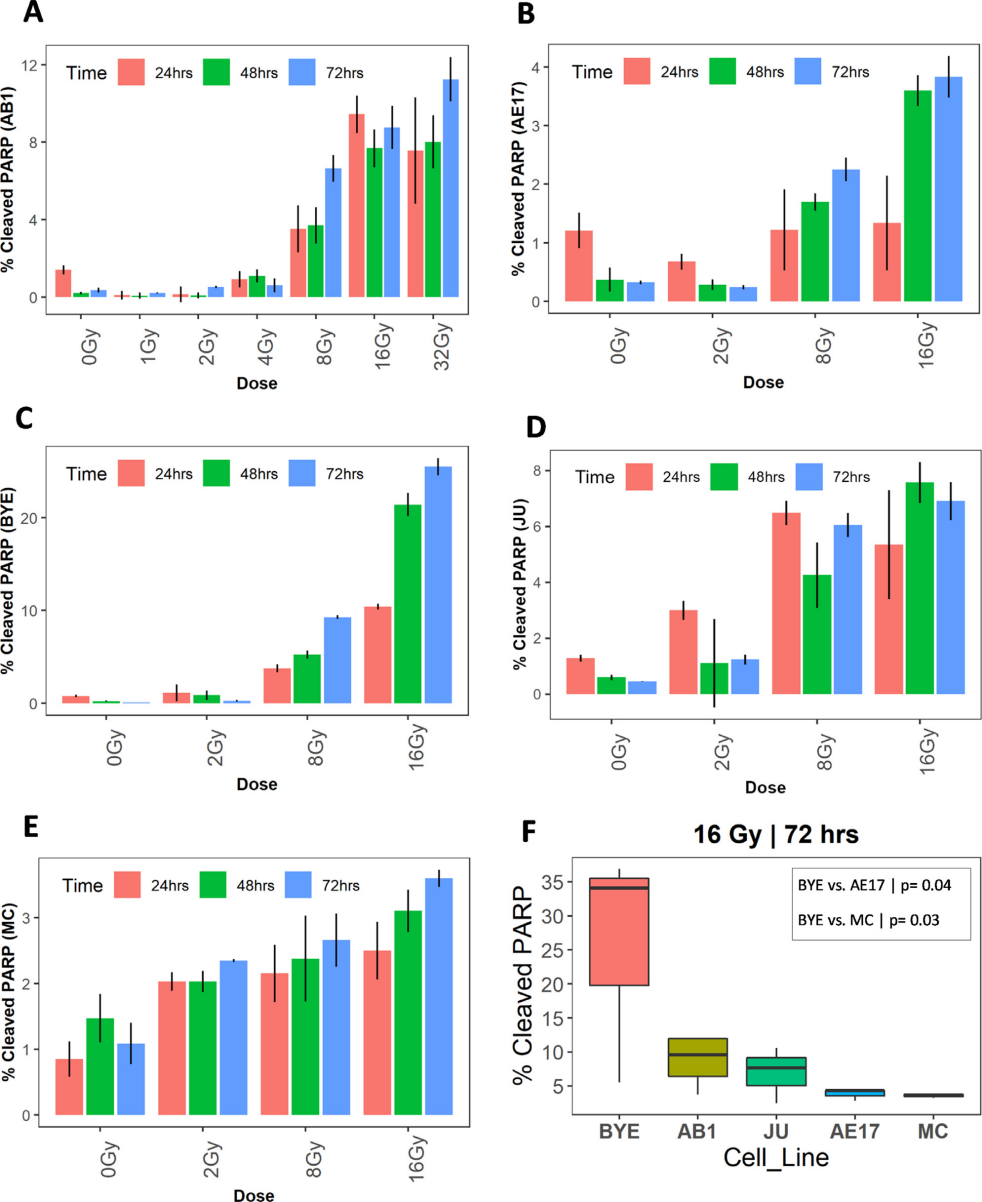
Apoptosis levels after radiation therapy differ between murine and human mesothelioma cell lines. A-E, Patterns of apoptosis of AB1, AE17, BYE, and MC cells, respectively. F, Comparison of the level of apoptosis among murine and human cell lines with 16 Gy at 72 hours. Data are presented as mean ± standard error. One-way analysis of variance for multiple group comparison with Tukey adjustment for pairwise comparison.

Next, we assessed apoptosis levels in response to radiation, through staining for the cleaved poly (ADP-ribose) polymerase (PARP-1) enzyme by flow cytometry (Fig. E5). Dose responses varied widely between cell lines; the human BYE and murine AB1 lines demonstrated approximately 10% of cells undergoing apoptosis 24 hours after a radiation dose of 16 Gy (increasing to 20% in the case of BYE after a further 24 hours) (Fig. 6A-C). However, apoptotic responses appeared lower in other cell lines studied, only reaching 4% to 5% of cells even 72 hours after treatment with 16 Gy in the AE17, JU, and MC lines (Fig. 6C, E). Comparison of apoptosis showed that the human cell line (BYE) had a higher proportion of cells undergoing apoptosis than did other cell lines (Fig. 6F).

### Discussion

RT is widely used to treat patients with mesothelioma.^45^ However, to our knowledge, the in vitro effects of photon radiation on cellular damage of mesothelioma cell lines and comparisons between effects in mouse and human cell lines have not previously been investigated intensively. In this study, we used a flow cytometry–based approach and clonogenic survival assays to examine the effects of photon radiation on the cellular damage of 2 well-characterized murine and 3 human mesothelioma cell lines. Our investigation assessed DNA damage, cell cycle, cell proliferation and survival, and apoptosis, which are the key indicators of cellular damage.^48^

As would be expected, we found that γ-H2AX increased with radiation dose in our studied cell lines, confirming a dose-response relationship. Maximum damage was induced by 8 Gy, above which γ-H2AX generation saturated. This finding was consistent with previous studies examining the phosphorylated histone H2AX by flow cytometry after exposure to ionizing radiation in human endothelial cells^49^ and several human cancer cell lines.^50^ γ-H2AX was strongly induced in all murine and human cell lines at 2 Gy compared with 0 Gy at 1 hour after radiation, except for the MC cell line. MC cells are very slow-growing compared with the other murine and human cell lines in our study, which may explain the low level of γ-H2AX at 2 Gy, because slow-growing cells are less sensitive to radiation^51^ than are rapidly proliferating cells.^52^^,^^53^ This implies that MC cells are resistant to DNA damage at low radiation doses but are radiosensitive at higher doses, because robust γ-H2AX levels were detected at 8 Gy.

Again, as expected, we showed that higher-dose radiation arrested cells at the G2/M phase, consistent with previous studies demonstrating G2/M arrest by radiation in leukemia and glioblastoma cell lines.^35^^,^^54^^,^^55^ In these studies, U87 glioblastoma cells were treated with 0 to 8 Gy, and G2/M arrest was induced by 4 Gy and 8 Gy at 12 and 24 hours after irradiation,^54^ whereas doses of 1.5 Gy to 6 Gy arrested leukemia cells at G2/M at 8 to 12 hours after irradiation. However, the arrest was reversible as cells re-entered the G0/G1 phase within 12 and 24 hours.^55^ Although we also observed G2/M arrest in our study, this did not occur at doses of 4 Gy or less nor at less than 24 hours after irradiation, which differs from the aforementioned studies in U87 glioblastoma and leukemia.^54^^,^^55^ Two possible reasons for the differences may have resulted from the limited dose ranges in our study. First, we treated the AB1 cell line with the full range of 1 to 32 Gy, whereas other cells lines were treated with only 2 Gy, 8 Gy, and 16 Gy. Therefore, we could only assess the cell-cycle phase distribution between larger dose increments, which allowed less accurate pinpointing of the exact dose at which G2/M phase arrest occurred. A second reason may have been the wider gap between each time point. It is feasible that the G2/M phase may have been arrested at a time point greater than 6 hours and less than 24 hours, which was not investigated in our study. However, a study by Murad et al^35^ showed that 10 Gy irradiation arrested T98G glioblastoma cells in G2/M from 24 hours, similar to our finding in murine mesothelioma cell lines. Interestingly, G2/M phase arrest was not observed with human cell lines at 2, 8, or 16 Gy, although there was disappearance of the G0/G1 population (BYE, JU, and MC) with 8 Gy and 16 Gy at 72 hours. This was consistent with a previous study in the U87-sph glioblastoma cell line demonstrating no cell-cycle arrest regardless of dose.^55^ The possible mechanism may be largely related to the protein Cdc25 (a protein inducing radioresistance) and the ATM/Chk1 signaling pathway. Previous studies have shown that the activation of the ATM/Chk1 pathway could inhibit Cdc25c activation, thereby preventing cell-cycle arrest at the G2/M phase.^54^^,^^56^, ^57^, ^58^ A recent study reported radiation-induced expression of an autophagy-related protein, BECN1, a key mediator of the G2/M transition. Disruption of BECN1 using autophagy inhibitor 3-methyladenine sensitized cells to G2/M arrest after irradiation through the prevention of phosphorylation of Cdc25c.^59^ Therefore, γ-radiation may have activated ATM/Chk1 and upregulated BECN1 in our study, which interfered with Cdc25c in human cell lines, leading to unobservable G2/M arrest. Further experiments investigating ATM/Chk1, Cdc25, and BECN1 are required to validate this hypothesis. Converse to reports in the literature,^60^^,^^61^ we did not observe G0/G1 arrests at the proposed time points in our study because the proportion of G0/G1 cells was not higher than in the untreated group. Cell-cycle arrest after radiation, especially in G2/M, gives sufficient time for cells to repair DNA. In our study, we found that a dose of 8 Gy temporarily arrested the AB1 cells at G2/M at 24 hours after irradiation. However, cells returned to normal levels with an increased G0/G1 peak at 48 and 72 hours. Doses greater than 8 Gy completely arrested cells at G2/M, suggesting the DNA damage induced by a dose ≥8 Gy is too extensive and hence permanently arrested cells at G2/M.

We also characterized DNA repair kinetics by modeling the decrease in γ-H2AX over time after photon irradiation. Gamma-H2AX is a DNA repair protein attracted to sites of DNA damage as the cells are arresting, thereby providing sufficient time for cellular repair.^48^ Previous studies showed γ-H2AX is a reliable marker to measure DNA double-stranded break repair.^62^, ^63^, ^64^, ^65^ We have demonstrated that murine and human mesothelioma cell lines repaired DNA effectively at lower radiation doses. However, the level of γ-H2AX remains higher after receiving 8 Gy compared with 1 Gy or 2 Gy. Temporal differences in γ-H2AX fluorescence intensity between low and high radiation doses may therefore suggest different cellular responses of mesothelioma cell lines to low- and high-dose radiation in our study. One explanation may be development of radioresistance by cells after low-dose RT, because they can repair the DNA more effectively than at high doses; this was also described in a review by Borrego-Soto et al.^51^ Alternatively, differences could be accounted for by less efficient DNA repair at higher doses; prolonged elevated expression of γ-H2AX, compared with lower doses, indicated excess acute and chronic toxicity induced by radiation, thereby impairing DNA repair machinery.^65^ Higher dose RT may severely affect nonhomologous end joining (NHEJ) (a key pathway of double-strand-break repair) via the suppression of NHEJ proteins such as XRCC4 and DNA Lig3 and Lig4, which are critical to forming a complex to ligate the broken ends during the NHEJ process. Decreases in XRCC4 and DNA Lig3 and Lig4 are associated with a decline in NHEJ efficiency and fidelity,^66^ and impaired NEHJ has been reported after irradiation in previous studies using myeloma cell lines.^67^ Therefore, the NEHJ pathway may have been badly disrupted in our human cells after higher doses of γ-irradiation. However, owing to the technical difficulties with multifraction irradiation in vitro*,* it is still difficult to conclude whether this phenomenon will occur in in vivo, or indeed, in the clinic, where fractionated RT is normally used; further research is therefore warranted. Moreover, by fitting multiple doses in a joint model of all cell lines, DNA repair was similar in all the studied cell lines, suggesting that this repair pattern occurs in both animal and human cells.

The most important measurable outcomes after radiation treatment in our study were cell proliferation, cell death (apoptosis), and cell survival as assessed by clonogenic survival assay. Our finding demonstrated that although lower-dose radiation (1-4 Gy) did not inhibit cell proliferation, high doses (16-32 Gy) reduced proliferation almost completely from 24 hours in murine cells, findings similar to those from Yao et al^68^ demonstrating a significant decrease in cell proliferation with 20 Gy at 24 hours. However, a study by Li et al^69^ showed low-dose radiation of 50, 75, and 100 mGy significantly inhibited cell proliferation of PC-3 cells compared with sham controls. In that study, low-dose radiation arrested cells at S and G2/M, differing from our findings because we did not observe G2/M arrest or inhibition of cell proliferation at low doses; this can be explained by different cell lines having differing sensitivities to radiation, and this is also likely to mainly be a factor resulting from different proliferation rates between cell lines, although there are also likely to be other more nuanced factors at play, such as the ability to repair DNA damage. Cell proliferation at 72 hours after irradiation showed the proliferation rate to be similar among all studied cell lines, because the slopes between cell and time were not significant. It is interesting to note that sham-irradiated MC human cells took up only 15% of BrdU in 1 hour pulse labeling, compared with approximately 45% BrdU uptake for murine cell lines. Therefore, if we examined cell proliferation at specific low doses, that is, 2 Gy at earlier time points after irradiation (eg, 1 or 6 hours), we would see the difference between MC and other cell lines. However, that difference might not suggest the inhibitory effect of radiation but the slow-growing nature of the MC cell line. It is interesting to note that decreased cell proliferation was clearly seen in all human cell lines from 8 Gy at 24, 48, and 72 hours, even though our data did not show significant cell-cycle arrest in G2/M, albeit reduction of the G0/G1 population in human cell lines 48 to 72 hours after doses ≥8 Gy were still observed. It may be that G2/M arrest peaks were not observed within the confines of the time points selected in our assay—for example, occurring between 6 to 24 hours. One further explanation is that these human cell lines (particularly BYE, which did not divide after higher-dose irradiation at 48 and 72 hours) may have undergone intrinsic apoptosis in a P53-dependent manner and did not require prior cell-cycle arrest.^70^,^71^^,^^72^ P53 activated by RT can directly trigger the release of proapoptotic genes such as p53‐upregulated modulator of apoptosis (PUMA) and BCL2‐associated X protein (BAX), which subsequently activate caspase-7, -9, and -3 to induce intrinsic apoptosis.^13^ Alternatively, human cell lines that ceased proliferation may have died through other cell death pathways not investigated in this study, such as necrosis, ecroptosis, and ferroptosis autophagy, independently of cell-cycle arrest.^69^^,^^73^ Further investigation is required to clarify the actual death mechanism. In addition to cell proliferation, cell survival probability decreased with increasing dose (1-8 Gy), with 8 Gy completely inhibiting colony formation in all studied cell lines. Interestingly, the α/β ratio of human cell lines was smaller than murine cell lines, indicating that human cells in our study were more sensitive to a change of dose per fraction than were murine cell lines. Our findings showed a smaller α/β ratio relative to that reported in a previous study,^74^ suggesting heterogeneity of response to radiation in human mesothelioma cell lines.

We found the AB1 and BYE human mesothelioma cell lines displayed more apoptosis between 8 Gy, 16 Gy, and 32 Gy, with a time dependence. Our finding is consistent with previous studies demonstrating the apoptotic sensitivity of colorectal cancer lines and human lung cancer.^75^^,^^76^ However, far lower levels of apoptosis were detected in other cell lines including AE17, JU, and MC. Previously, several glioblastoma cell lines have shown no apoptosis after treatment with 20 Gy.^68^ Interestingly, a previous study in H661 and H520 non-small cell lung cancer lines observed greater apoptosis after radiation at 144 hours.^77^ The latest time point in our study was 72 hours; hence, it is possible that we might have seen more apoptosis in AE17, JU, and MC if we had extended the observation time. To induce apoptosis, the phosphorylation of P53 and activation of caspase-3 and -7 after radiation are required.^13^ Therefore, these signals might not yet have been fully activated in AE17, JU, and MC cell lines in our study. Future studies may consider investigating other regulators of apoptosis such as cleaved caspase-3 and -7 or upregulation of Bax/Bcl-2 to validate our current finding.^13^^,^^76^ BRCA1-associated protein 1 gene (BAP1) mutations have been linked to early onset of MPM and resistance to chemotherapy.^78^ In renal cell carcinoma, knockout of BAP1 resulted in sensitization to apoptosis after γ-irradiation.^79^ The human cell lines used in our study were variably positive (MC line) or negative (JU and BYE) for BAP1, but this did not seem to be indicative of responses to irradiation in our study.

In conclusion, in murine and human mesothelioma cell lines, radiation was shown to induce DNA damage and G2/M arrest, inhibit cell proliferation, and cause apoptosis in a defined and quantifiable manner. Compared with lower doses (1-2 Gy), a dose of 8 Gy demonstrated significantly increased damaging effects. Considering DNA damage repair and the apoptotic mechanism of cells at the cellular level after different doses of irradiation, it can be concluded that an approximate dose of 8 Gy may be an optimal hypofractionated dose to induce cellular damages in our studied cell lines and may be able to generate immunogenic cell death to activate local and systemic immune response for future radioimmunotherapy study in a mouse model. However, because only epithelioid human mesothelioma cell lines were studied, our observation does not necessarily apply for other nonepithelioid mesothelioma, which are typically more resistant to radiation. Additionally, we have identified a 72-hour postradiation time point at which increased inhibition of cell proliferation was consistently observed across several cell lines; this may be an ideal time for combination with immunotherapies (eg, anti-PD-L1), because response rates in mesothelioma are still generally low. Cytotoxic chemotherapy has been shown to improve response rates to anti-PD-L1 in mesothelioma^80^; RT may have the potential to perform a similar role, minus the systemic toxic effects observed with chemotherapy. The similarity between mouse and human cell lines, particularly cell proliferation and cell survival after irradiation with 8 Gy or above, suggests mouse cell lines provide a good model that is broadly analogous to human cell lines, although human cell lines exhibit variable responsiveness to radiation, as seen in clinical practice. However, it is important to note that significant DNA damage induced by our prescribed RT dose (eg, 8 Gy) may not be similarly observed with in vivo mesothelioma tumors. There may be different effects when cells are irradiated in the context of intratumoral stromal cells such as cancer-associated fibroblasts, endothelial cells, suppressive immune cells, regulatory cytokines, the extracellular matrix, and regions of hypoxia. Stromal cells and hypoxia may drive the resistance of mesothelioma to RT compared with our current in vitro study, where only single cell suspensions were irradiated.

Given that the recent approval of nivolumab plus ipilimumab as first-line treatment for mesothelioma significantly improved progression-free survival only in patients with nonepithelioid cancer,^4^ improved therapies are still required. RT is a potential partner for immune checkpoint blockade through induction of immunogenic cell death, potentially converting tumors from being immunologically “cold” to “hot,” critical for immunotherapy responses.^13^ The work we have described here represents an initial step toward developing an in vivo preclinical model system in which robust studies into the optimal ways to combine radiation and immunotherapy can be run. We have shown our murine mesothelioma cell lines to be broadly similar to their human equivalents in regard to their responses to increasing photon radiation dose, mainly expressed as DNA damage, decreased cell proliferation, and survival with a dose of 6 or 8 Gy. These clinically relevant hypofractionated doses may now be taken forward to preclinical radioimmunotherapy studies, with several further aspects now requiring investigation before generation of a fully optimized murine model; these will include scheduling of multiple RT fractions, the interaction of treatment with the full tumor microenvironment rather than just cancer cells (particularly in the context of an intact host immune system), and, ultimately, how best to combine with immune checkpoint inhibitors in an effort to unlock their full potential.

## Appendix Supplementary materials

Image, application 1

Image, application 2

Image, application 3

Image, application 4

## Footnotes

Supplementary material associated with this article can be found in the online version at doi:10.1016/j.adro.2022.101013.
