# Supplementary material for: Effects of Photon Radiation on DNA Damage, Cell Proliferation, Cell Survival, and Apoptosis of Murine and Human Mesothelioma Cell Lines
Source: Adv Radiat Oncol. 2022 Jul 21;7(6):101013. doi: 10.1016/j.adro.2022.101013 (PMC9677206; doi:10.1016/j.adro.2022.101013)
Supplement: Supplementary file 1 [file mmc1.docx]

**Supplementary Tables**

***Table 1: Characteristics of murine and human mesothelioma cell lines***

| **Cell line** | **Source** | **# Passage** | **Sex** | **Age at diagnosis** | **Histology** |
| --- | --- | --- | --- | --- | --- |
| AB1 | BALB/c | 24 | F | NA | NA |
| AE17 | C57BL/6 | 12 | F | NA | NA |
| BYE | Human | 10 | M | 75 | Epithelioid |
| MC | Human | 15 | M | 70 | Epithelioid |
| JU | Human | 67 | M | 63 | Not specified |

***Table 2. Pairwise comparison of ED50 parameter of log-logistic regression model between studied cell lines***

| **Pair-wise** | **Difference in coefficient** | **p-value** |
| --- | --- | --- |
| AB1-AE17 | -0.49 | 0.85 |
| AB1-BYE | -0.55 | 0.26 |
| AB1-JU | -1.38 | 0.06 |
| **AB1-MC** | **-3.30** | **0.01** |
| AE17-BYE | -0.06 | 0.98 |
| AE17-JU | -0.89 | 0.74 |
| AE17-MC | -2.82 | 0.32 |
| BYE-JU | -0.83 | 0.17 |
| **BYE-MC** | **-2.76** | **0.01** |
| JU-MC | -1.93 | 0.13 |

***Table 3: p-value for pairwise comparison of cell cycle for AB1, AE17, BYE, JU and MC***

| **3A (AB1)** | | | 1 Gy | 2 Gy | 4 Gy | 8 Gy | 16 Gy | 32 Gy |
| --- | --- | --- | --- | --- | --- | --- | --- | --- |
| 1H | G1/G0 | 0 Gy | 0.817 | 0.996 | 0.997 | 0.788 | 0.973 | 0.977 |
|  |  | 1 Gy | - | 0.985 | 0.967 | 1 | 0.29 | 0.303 |
|  |  | 2 Gy | - | - | 1 | 0.983 | 0.747 | 0.763 |
|  |  | 4 Gy | - | - | - | 0.961 | 0.728 | 0.745 |
|  |  | 8 Gy | - | - | - | - | 0.231 | 0.243 |
|  |  | 16 Gy | - | - | - | - | - | 1 |
|  | G2/M | 0 Gy | 0.7112 | 0.7112 | 0.999 | 0.895 | 1 | 0.993 |
|  |  | 1 Gy | - | 0.996 | 0.827 | 0.121 | 0.612 | 0.288 |
|  |  | 2 Gy | - | - | 0.478 | 0.138 | 0.282 | 0.102 |
|  |  | 4 Gy | - | - | - | 0.672 | 0.999 | 0.931 |
|  |  | 8 Gy | - | - | - | - | 0.884 | 0.997 |
|  |  | 16 Gy | - | - | - | - | - | 0.994 |
| 6H | G1/G0 | 0 Gy | 0.998 | 0.622 | 0.321 | 0.304 | 0.987 | 0.999 |
|  |  | 1 Gy | - | 0.876 | 0.606 | 0.584 | 0.999 | 0.983 |
|  |  | 2 Gy | - | - | 0.999 | 0.999 | 0.926 | 0.397 |
|  |  | 4 Gy | - | - | - | 1 | 0.669 | 0.149 |
|  |  | 8 Gy | - | - | - | - | 0.646 | 0.14 |
|  |  | 16 Gy | - | - | - | - | - | 0.922 |
|  | G2/M | 0 Gy | 0.48 | 0.075 | 0.066 | 0.86 | 0.95 | 0.93 |
|  |  | 1 Gy | - | 0.421 | 0.377 | 0.543 | 0.458 | 0.186 |
|  |  | 2 Gy | - | - | 0.982 | 0.142 | 0.109 | 0.614 |
|  |  | 4 Gy | - | - | - | 0.107 | 1 | 0.364 |
|  |  | 8 Gy | - | - | - | - | 0.885 | 0.441 |
|  |  | 16 Gy | - | - | - | - | - | 1 |
| 24H | G1/G0 | 0 Gy | 0.999 | 0.999 | 0.999 | 0.008 | 0.002 | 0.003 |
|  |  | 1 Gy | - | 0.999 | 0.999 | 0.043 | 0.001 | 0.001 |
|  |  | 2 Gy | - | - | 0.999 | 0.058 | 0.001 | 0.002 |
|  |  | 4 Gy | - | - | - | 0.044 | 0.001 | 0.001 |
|  |  | 8 Gy | - | - | - | - | 0.455 | 0.598 |
|  |  | 16 Gy | - | - | - | - | - | 0.999 |
|  | G2/M | 0 Gy | 0.997 | 0.991 | 0.86 | 0.117 | 0.001 | 0.007 |
|  |  | 1 Gy | - | 0.999 | 0.993 | 0.32 | 0.005 | 0.023 |
|  |  | 2 Gy | - | - | 0.997 | 0.382 | 0.01 | 0.029 |
|  |  | 4 Gy | - | - | - | 0.613 | 0.01 | 0.053 |
|  |  | 8 Gy | - | - | - | - | 0.278 | 0.71 |
|  |  | 16 Gy | - | - | - | - | - | 0.983 |
| 48H | G1/G0 | 0 Gy | 0.563 | 0.974 | 0.734 | 0.798 | 0 | 0 |
|  |  | 1 Gy | - | 0.96 | 0.999 | 0.048 | 0 | 0 |
|  |  | 2 Gy | - | - | 0.996 | 0.289 | 0 | 0 |
|  |  | 4 Gy | - | - | - | 0.068 | 0 | 0 |
|  |  | 8 Gy | - | - | - | - | 0 | 0 |
|  |  | 16 Gy | - | - | - | - | - | 0.946 |
|  | G2/M | 0 Gy | 0.995 | 0.999 | 0.999 | 0.966 | 0 | 0 |
|  |  | 1 Gy | - | 0.999 | 0.986 | 0.999 | 0 | 0 |
|  |  | 2 Gy | - | - | 0.997 | 0.998 | 0 | 0 |
|  |  | 4 Gy | - | - | - | 0.92 | 0 | 0 |
|  |  | 8 Gy | - | - | - | - | 0 | 0 |
|  |  | 16 Gy | - | - | - | - | - | 0.001 |
| 72H | G1/G0 | 0 Gy | 0.922 | 0.828 | 0.946 | 0.81 | 0.64 | 0.006 |
|  |  | 1 Gy | - | 0.999 | 0.999 | 0.203 | 0.005 | 0.001 |
|  |  | 2 Gy | - | - | 0.999 | 0.13 | 0.003 | 0.001 |
|  |  | 4 Gy | - | - | - | 0.194 | 0.004 | 0 |
|  |  | 8 Gy | - | - | - | - | 0.474 | 0.066 |
|  |  | 16 Gy | - | - | - | - | - | 0.884 |
|  | G2/M | 0 Gy | 0.999 | 0.999 | 0.999 | 0.974 | 0.106 | 0.017 |
|  |  | 1 Gy | - | 1 | 0.999 | 0.893 | 0.058 | 0.009 |
|  |  | 2 Gy | - | - | 1 | 0.918 | 0.067 | 0.01 |
|  |  | 4 Gy | - | - | - | 0.91 | 0.046 | 0.006 |
|  |  | 8 Gy | - | - | - | - | 0.336 | 0.061 |
|  |  | 16 Gy | - | - | - | - | - | 0.95 |

| **3B (AE17)** | | G1/G0 | | | G2/M | | |
| --- | --- | --- | --- | --- | --- | --- | --- |
|  |  | 2 Gy | 8 Gy | 16 Gy | 2 Gy | 8 Gy | 16 Gy |
| 1H | 0 | 0.249 | 0.027 | 0.212 | 0.888 | 0.562 | 0.401 |
|  | 2 Gy | - | 0.428 | 0.43 | - | 0.244 | 0.782 |
|  | 8 Gy | - | - | 0.677 | - | - | 0.066 |
| 6H | 0 | 1 | 0.013 | 0.249 | 1 | 0.53 | 0.249 |
|  | 2 Gy | - | 0.25 | 1 | - | 0.249 | 0.105 |
|  | 8 Gy | - | - | 1 | - | - | 1 |
| 24H | 0 | 0.098 | 0.044 | 0.000 | 0.999 | 0.018 | 0.000 |
|  | 2 Gy | - | 0.941 | 0.001 | - | 0.019 | 0.000 |
|  | 8 Gy | - | - | 0.001 | - | - | 0.000 |
| 48H | 0 | 0.778 | 0.202 | 0.004 | 0.968 | 0.861 | 0.185 |
|  | 2 Gy | - | 0.617 | 0.014 | - | 0.986 | 0.33 |
|  | 8 Gy | - | - | 0.079 | - | - | 0.486 |
| 72H | 0 | 0.26 | 0.093 | 0.003 | 0.607 | 0.967 | 0.015 |
|  | 2 Gy | - | 0.875 | 0.04 | - | 0.847 | 0.003 |
|  | 8 Gy | - | - | 0.117 | - | - | 0.008 |

| **3C (BYE)** | | G1/G0 | | | G2/M | | |
| --- | --- | --- | --- | --- | --- | --- | --- |
|  |  | 2 Gy | 8 Gy | 16 Gy | 2 Gy | 8 Gy | 16 Gy |
| 1H | 0 | NS | NS | NS | NS | NS | NS |
|  | 2 Gy | - | NS | NS | - | NS | NS |
|  | 8 Gy | - | - | NS | - | - | NS |
| 6H | 0 | NS | NS | NS | NS | NS | NS |
|  | 2 Gy | - | NS | NS | - | NS | NS |
|  | 8 Gy | - | - | NS | - | - | NS |
| 24H | 0 | 0.992 | 0.133 | 0.018 | NS | NS | NS |
|  | 2 Gy | - | 0.192 | 0.027 | - | NS | NS |
|  | 8 Gy | - | - | 0.53 | - | - | NS |
| 48H | 0 | 0.985 | 0.173 | 0.03 | NS | NS | NS |
|  | 2 Gy | - | 0.107 | 0.018 | - | NS | NS |
|  | 8 Gy | - | - | 0.615 | - | - | NS |
| 72H | 0 | 0.982 | 0.046 | 0.026 | NS | NS | NS |
|  | 2 Gy | - | 0.027 | 0.016 | - | NS | NS |
|  | 8 Gy | - | - | 0.976 | - | - | NS |

| **3D (JU)** | | G1/G0 | | | G2/M | | |
| --- | --- | --- | --- | --- | --- | --- | --- |
|  |  | 2 Gy | 8 Gy | 16 Gy | 2 Gy | 8 Gy | 16 Gy |
| 1H | 0 | NS | NS | NS | NS | NS | NS |
|  | 2 Gy | - | NS | NS | - | NS | NS |
|  | 8 Gy | - | - | NS | - | - | NS |
| 6H | 0 | NS | NS | NS | NS | NS | NS |
|  | 2 Gy | - | NS | NS | - | NS | NS |
|  | 8 Gy | - | - | NS | - | - | NS |
| 24H | 0 | NS | NS | NS | NS | NS | NS |
|  | 2 Gy | - | NS | NS | - | NS | NS |
|  | 8 Gy | - | - | NS | - | - | NS |
| 48H | 0 | 0.698 | 0.016 | 0.001 | NS | NS | NS |
|  | 2 Gy | - | 0.076 | 0.003 | - | NS | NS |
|  | 8 Gy | - | - | 0.167 | - | - | NS |
| 72H | 0 | 0.02 | 0 | 0 | NS | NS | NS |
|  | 2 Gy | - | 0.001 | 0 | - | NS | NS |
|  | 8 Gy | - | - | 0.02 | - | - | NS |

| **3E (MC)** | | G1/G0 | | | G2/M | | |
| --- | --- | --- | --- | --- | --- | --- | --- |
|  |  | 2 Gy | 8 Gy | 16 Gy | 2 Gy | 8 Gy | 16 Gy |
| 1H | 0 | NS | NS | NS | NS | NS | NS |
|  | 2 Gy | - | NS | NS | - | NS | NS |
|  | 8 Gy | - | - | NS | - | - | NS |
| 6H | 0 | NS | NS | NS | NS | NS | NS |
|  | 2 Gy | - | NS | NS | - | NS | NS |
|  | 8 Gy | - | - | NS | - | - | NS |
| 24H | 0 | NS | NS | NS | NS | NS | NS |
|  | 2 Gy | - | NS | NS | - | NS | NS |
|  | 8 Gy | - | - | NS | - | - | NS |
| 48H | 0 | NS | NS | NS | NS | NS | NS |
|  | 2 Gy | - | NS | NS | - | NS | NS |
|  | 8 Gy | - | - | NS | - | - | NS |
| 72H | 0 | NS | NS | NS | NS | NS | NS |
|  | 2 Gy | - | NS | NS | - | NS | NS |
|  | 8 Gy | - | - | NS | - | - | NS |

***Table 4: Summary of p-value for the pairwise comparisons for DNA repair between different doses of radiation***

| **(AB1)** | 2 Gy | 4 Gy | 8 Gy | 16 Gy | 32 Gy |
| --- | --- | --- | --- | --- | --- |
| 1 Gy | 0.999 | 0.59 | 0.01 | <0.01 | <0.01 |
| 2 Gy | - | 0.78 | 0.01 | <0.01 | <0.01 |
| 4 Gy | - | - | 0.25 | <0.01 | <0.01 |
| 8 Gy | - | - | - | <0.01 | 0.10 |
| 16 Gy | - | - | - | - | 0.87 |

| **(AE17)** | 8 Gy | 16 Gy | **(BYE)** | 8 Gy | 16 Gy |
| --- | --- | --- | --- | --- | --- |
| 2 Gy | <0.01 | <0.01 | 2 Gy | <0.01 | <0.01 |
| 8 Gy | - | <0.01 | 8 Gy | - | 0.18 |
| 16 Gy | - | - | 16 Gy | - | - |

| **(JU)** | 8 Gy | 16 Gy | **(MC)** | 8 Gy | 16 Gy |
| --- | --- | --- | --- | --- | --- |
| 2 Gy | <0.01 | <0.01 | 2 Gy | 0.01 | <0.01 |
| 8 Gy | - | 0.99 | 8 Gy | - | 0.44 |
| 16 Gy | - | - | 16 Gy | - | - |

***Table 5: Parameter estimates (α & β) for the cell survival curves***

|  | α (Gy^-1^) | β (Gy^-2^) | α/β ratio (Gy) |
| --- | --- | --- | --- |
| AB1 | 0.15 | 0.04 | 3.34 |
| AE17 | 0.25 | 0.05 | 4.79 |
| BYE | 0.11 | 0.06 | 1.76 |
| JU | 0.12 | 0.12 | 0.97 |
