# Supplementary material for: Effects of Photon Radiation on DNA Damage, Cell Proliferation, Cell Survival, and Apoptosis of Murine and Human Mesothelioma Cell Lines
Source: Adv Radiat Oncol. 2022 Jul 21;7(6):101013. doi: 10.1016/j.adro.2022.101013 (PMC9677206; doi:10.1016/j.adro.2022.101013)
Supplement: Supplementary file 2 [file mmc2.pptx]

## Slide 1
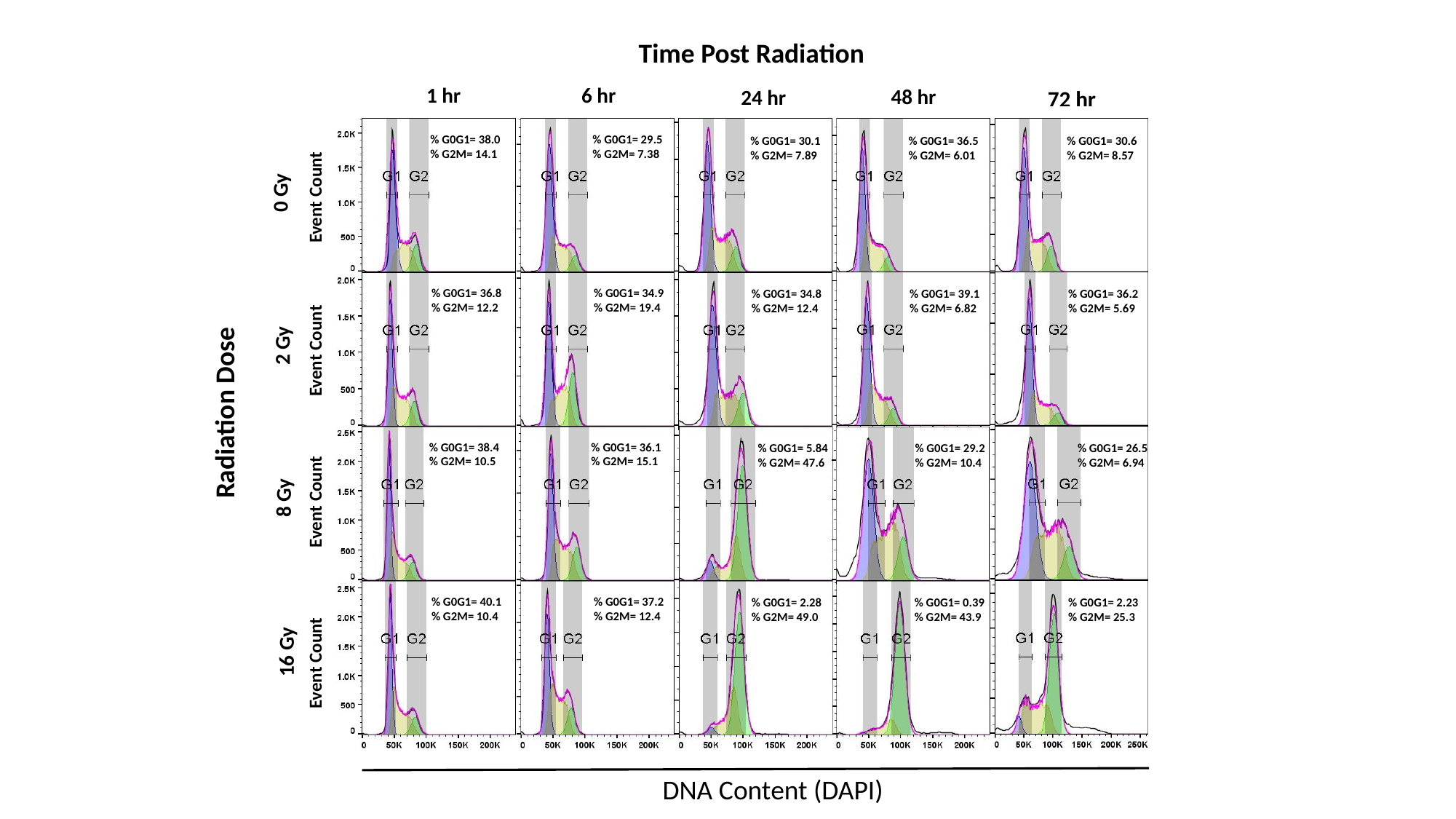

Time Post Radiation
% G0G1= 38.0
% G2M= 14.1
% G0G1= 29.5
% G2M= 7.38
% G0G1= 30.1
% G2M= 7.89
% G0G1= 30.6
% G2M= 8.57
% G0G1= 36.5
% G2M= 6.01
0 Gy
% G0G1= 36.8
% G2M= 12.2
% G0G1= 34.9
% G2M= 19.4
% G0G1= 34.8
% G2M= 12.4
% G0G1= 36.2
% G2M= 5.69
% G0G1= 39.1
% G2M= 6.82
Event Count
Radiation Dose
2 Gy
% G0G1= 38.4
% G2M= 10.5
% G0G1= 36.1
% G2M= 15.1
% G0G1= 5.84
% G2M= 47.6
% G0G1= 26.5
% G2M= 6.94
% G0G1= 29.2
% G2M= 10.4
Event Count
8 Gy
% G0G1= 40.1
% G2M= 10.4
% G0G1= 37.2
% G2M= 12.4
% G0G1= 2.28
% G2M= 49.0
% G0G1= 2.23
% G2M= 25.3
% G0G1= 0.39
% G2M= 43.9
Event Count
16 Gy
DNA Content (DAPI)
1 hr
6 hr
48 hr
24 hr
72 hr
Event Count

## Slide 2
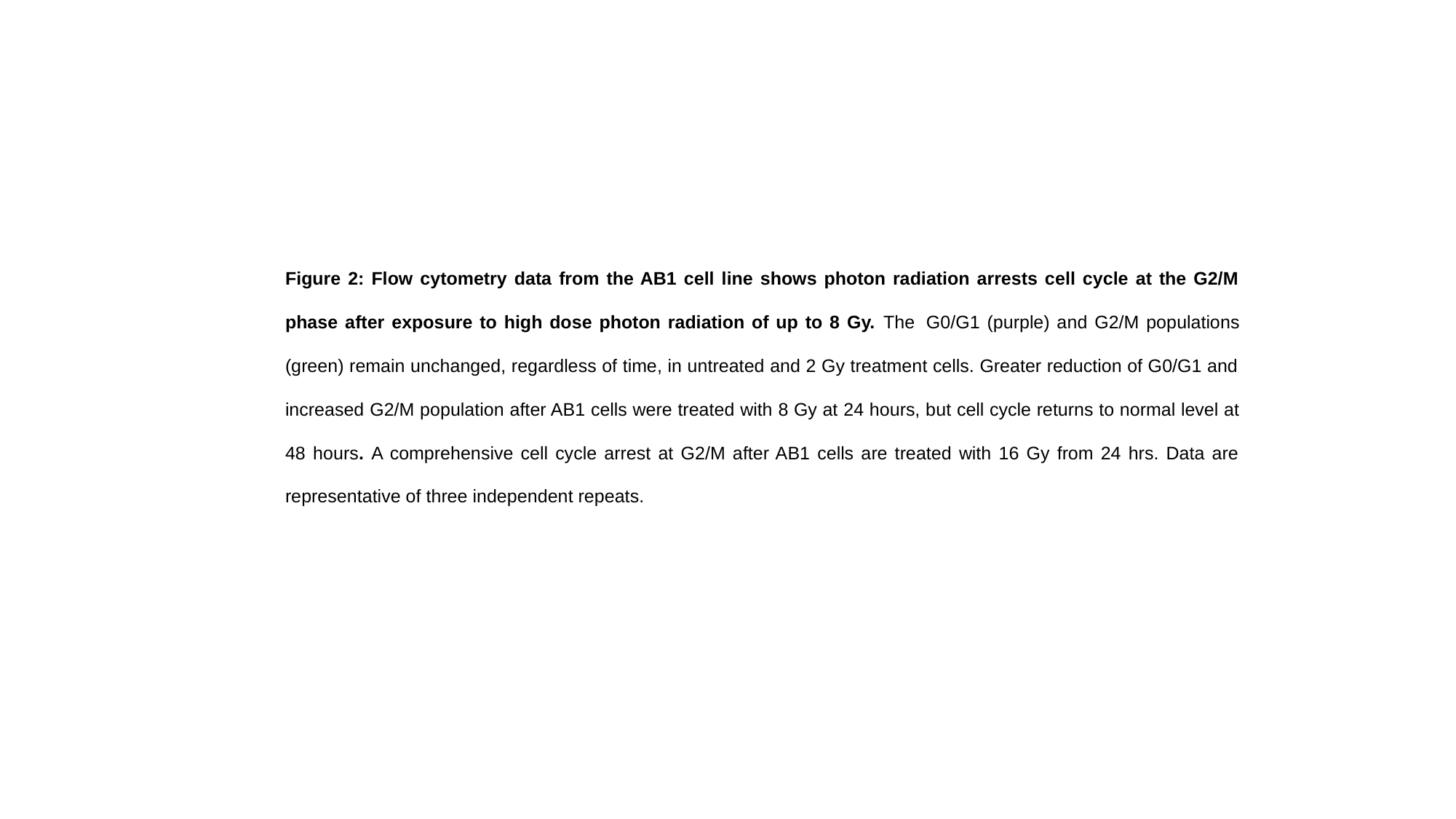

Figure 2: Flow cytometry data from the AB1 cell line shows photon radiation arrests cell cycle at the G2/M phase after exposure to high dose photon radiation of up to 8 Gy. The  G0/G1 (purple) and G2/M populations (green) remain unchanged, regardless of time, in untreated and 2 Gy treatment cells. Greater reduction of G0/G1 and increased G2/M population after AB1 cells were treated with 8 Gy at 24 hours, but cell cycle returns to normal level at 48 hours. A comprehensive cell cycle arrest at G2/M after AB1 cells are treated with 16 Gy from 24 hrs. Data are representative of three independent repeats.
