# Supplementary material for: Effects of Photon Radiation on DNA Damage, Cell Proliferation, Cell Survival, and Apoptosis of Murine and Human Mesothelioma Cell Lines
Source: Adv Radiat Oncol. 2022 Jul 21;7(6):101013. doi: 10.1016/j.adro.2022.101013 (PMC9677206; doi:10.1016/j.adro.2022.101013)
Supplement: Supplementary file 3 [file mmc3.pptx]

## Slide 1
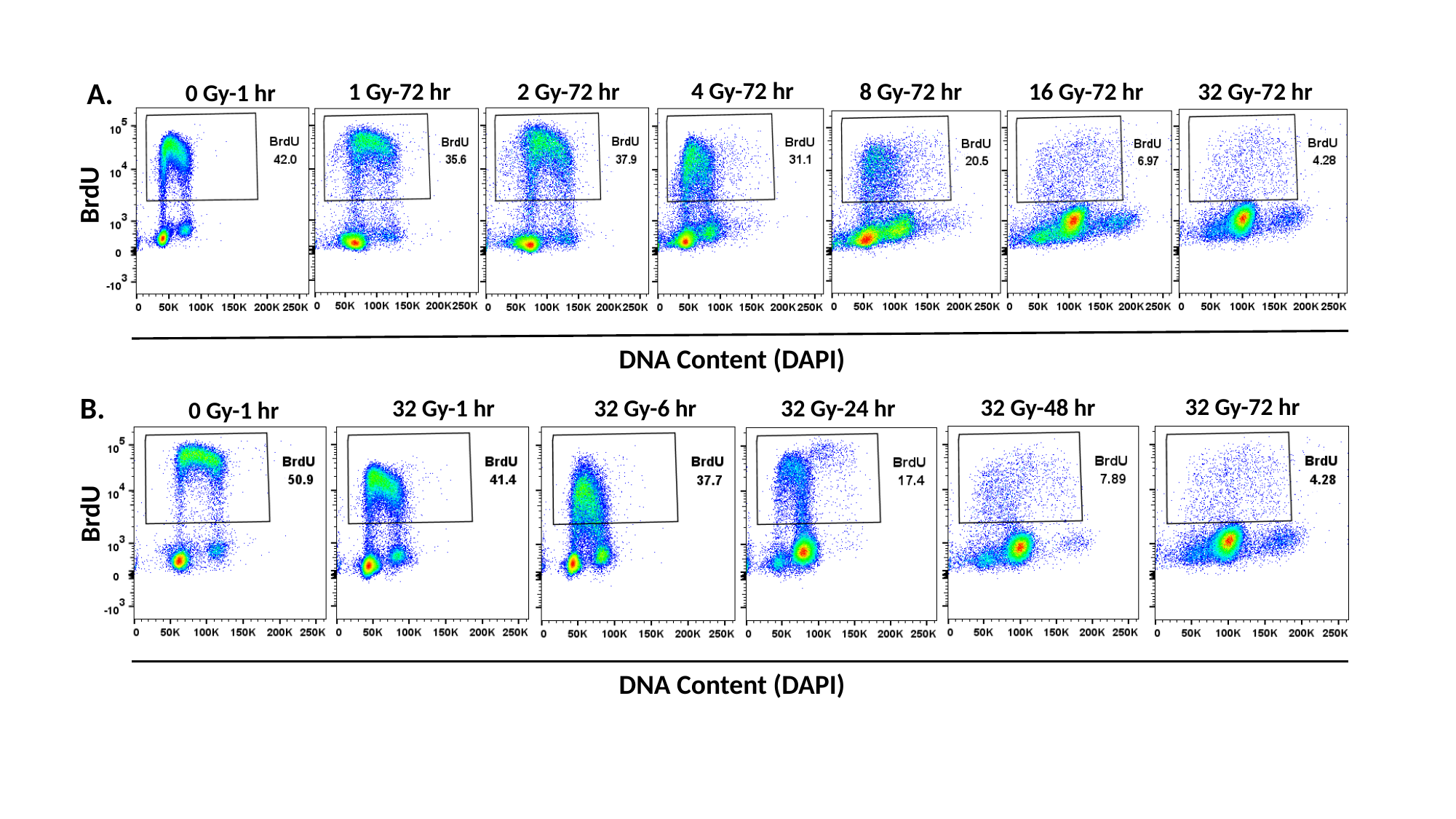

A.
4 Gy-72 hr
16 Gy-72 hr
32 Gy-72 hr
1 Gy-72 hr
2 Gy-72 hr
8 Gy-72 hr
0 Gy-1 hr
BrdU
DNA Content (DAPI)
B.
32 Gy-72 hr
32 Gy-48 hr
32 Gy-24 hr
32 Gy-1 hr
32 Gy-6 hr
0 Gy-1 hr
BrdU
DNA Content (DAPI)

## Slide 2
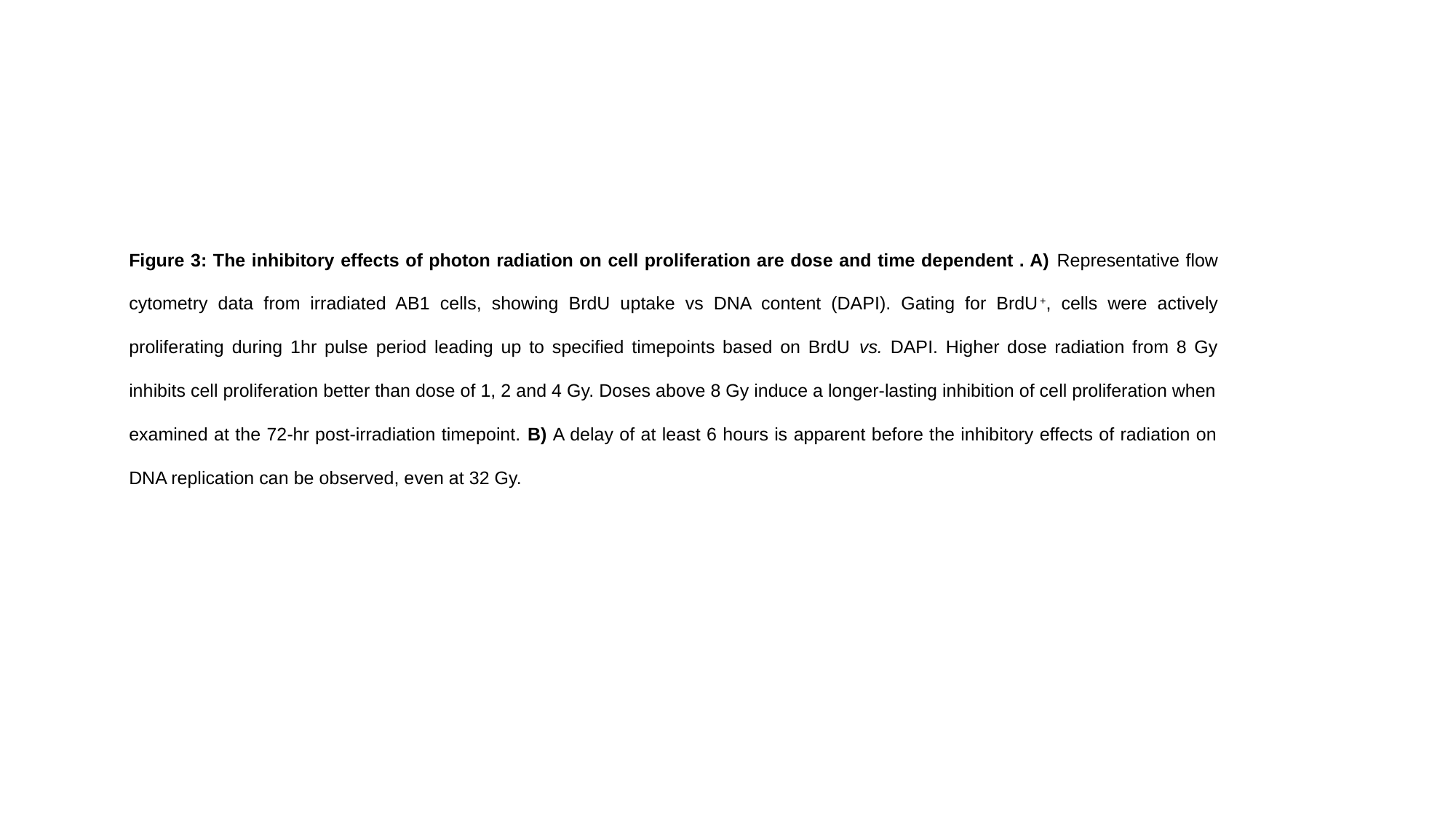

Figure 3: The inhibitory effects of photon radiation on cell proliferation are dose and time dependent . A) Representative flow cytometry data from irradiated AB1 cells, showing BrdU uptake vs DNA content (DAPI). Gating for BrdU+, cells were actively proliferating during 1hr pulse period leading up to specified timepoints based on BrdU vs. DAPI. Higher dose radiation from 8 Gy inhibits cell proliferation better than dose of 1, 2 and 4 Gy. Doses above 8 Gy induce a longer-lasting inhibition of cell proliferation when examined at the 72-hr post-irradiation timepoint. B) A delay of at least 6 hours is apparent before the inhibitory effects of radiation on DNA replication can be observed, even at 32 Gy.
