# Supplementary material for: Effects of Photon Radiation on DNA Damage, Cell Proliferation, Cell Survival, and Apoptosis of Murine and Human Mesothelioma Cell Lines
Source: Adv Radiat Oncol. 2022 Jul 21;7(6):101013. doi: 10.1016/j.adro.2022.101013 (PMC9677206; doi:10.1016/j.adro.2022.101013)
Supplement: Supplementary file 4 [file mmc4.docx]

**Supplementary Figures**

**
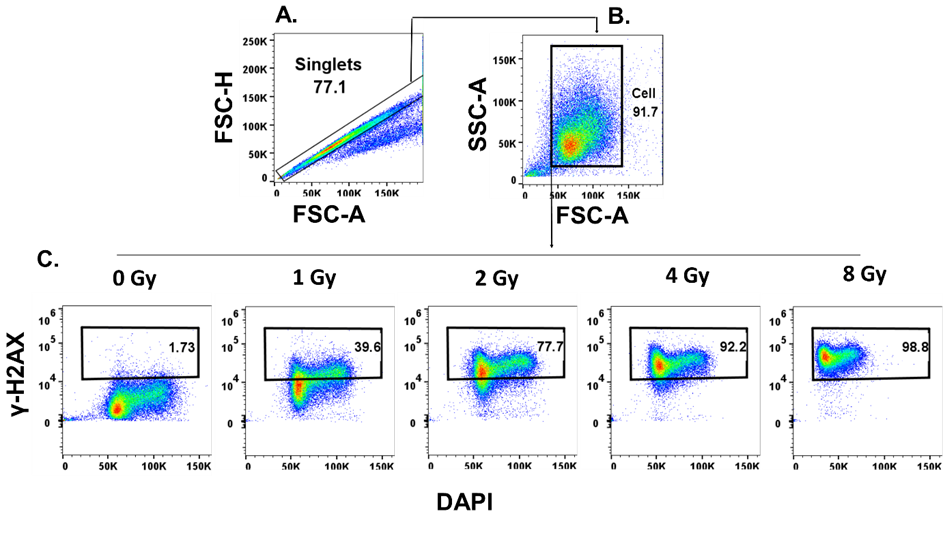
**

**Figure 1**: **Gating strategy for DNA damage analysis (γ-H2AX).** Representative data, showing one repeat using the AB1 cell line at 1 hour post-irradiation. **(A)** Single cells were gated based on FSC-H vs. FSC-A. **(B)** Debris were excluded based on SSC-A vs. FSC-A. **(C)** The level of γ-H2AX was gated based on γ-H2AX *vs*. DAPI. Gating for true γ-H2AX was based on sham-irradiated cells (0 Gy).


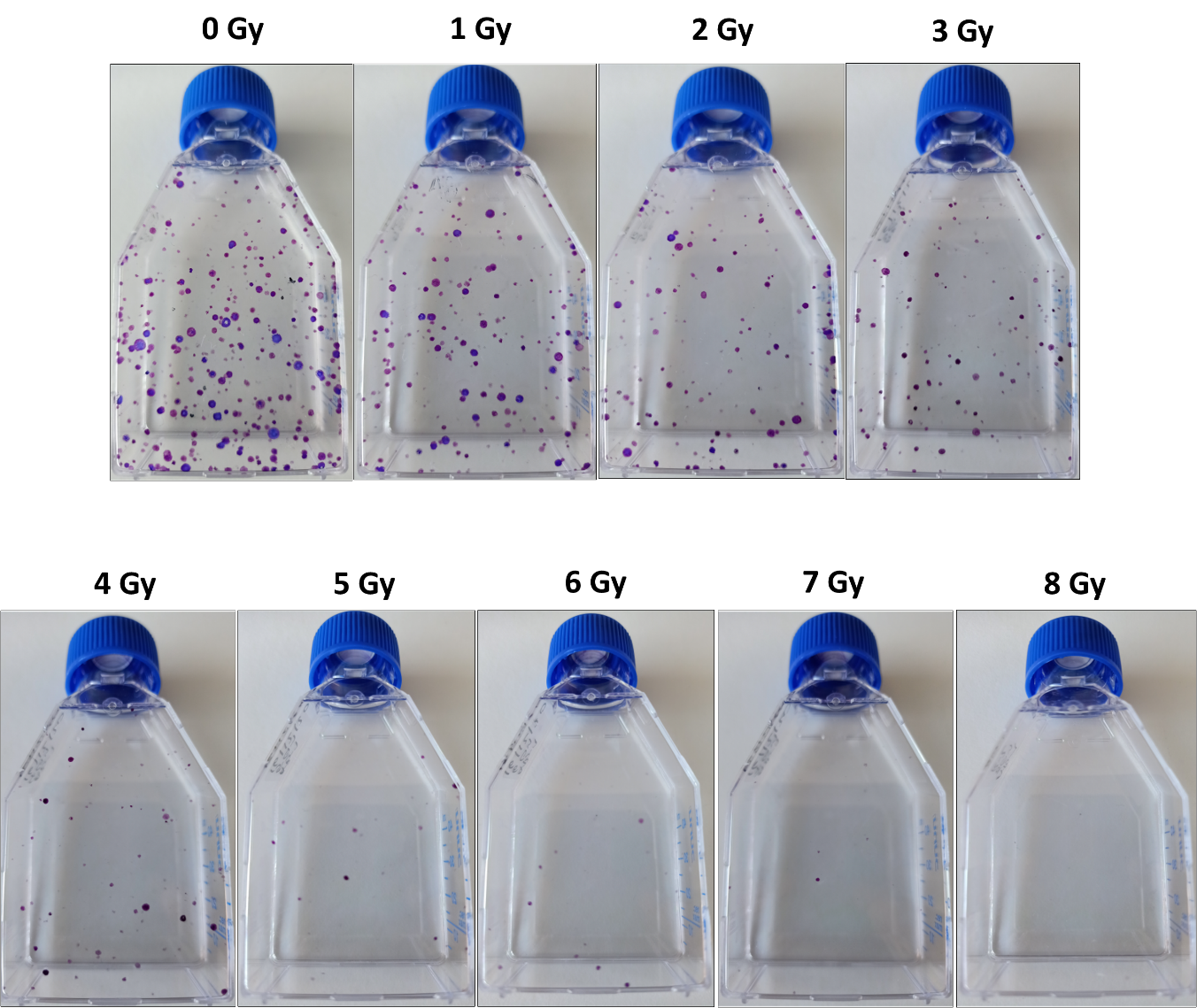


**Figure 4:** Representative cell survival of AB1 cell line.


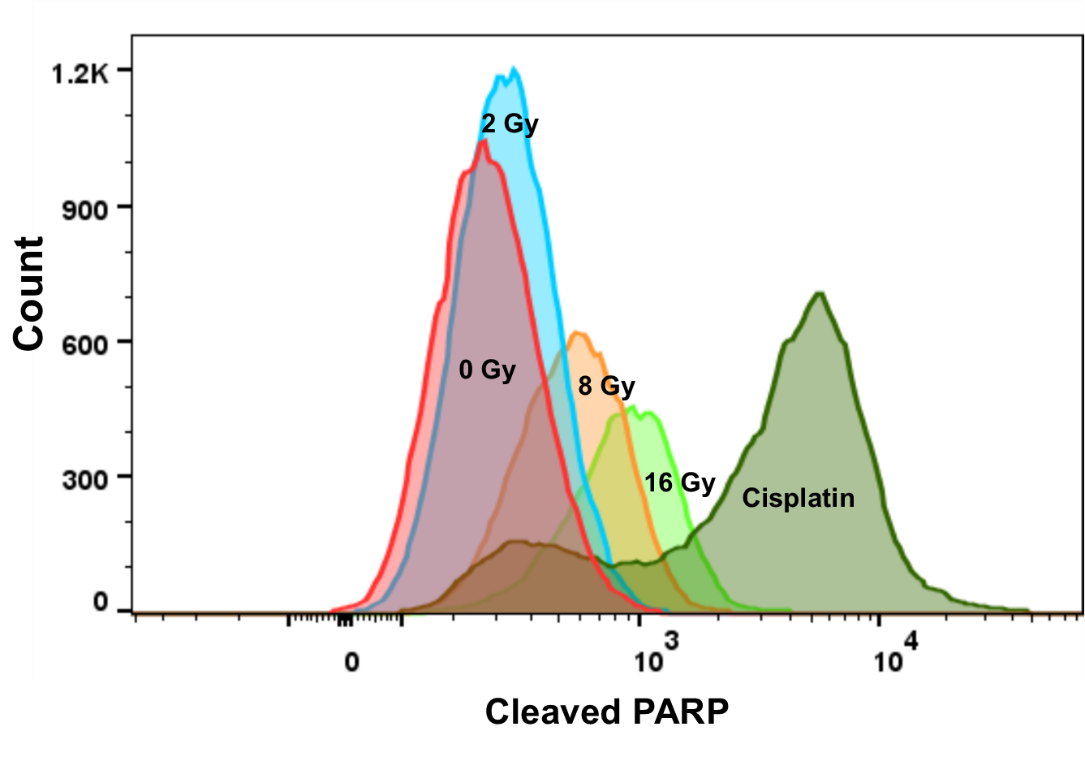


**Figure 5**: **Representative histogram plots from flow cytometry of cleaved PARP after treating with increasing doses of photon radiation**. Greater levels of cleaved PARP was observed with 16 Gy at 72 hours compared to dose of 0 Gy, 2 Gy and 8 Gy. Gates were set using un-irradiated (0 Gy) cells, above which cells were deemed as positive. Cisplatin was used as positive control for apoptosis and cleaved PARP expression
